# Ascending propriospinal modulation of thoracic sympathetic preganglionic neurons during lumbar locomotor activity

**DOI:** 10.3389/fncir.2026.1738731

**Published:** 2026-03-20

**Authors:** Lucia E. Dominguez-Rodriguez, Chioma V. Nwachukwu, Narjes Shahsavani, Juanita Garcia, Jeremy W. Chopek, Kristine C. Cowley

**Affiliations:** Department of Physiology and Pathophysiology, Rady Faculty of Health Sciences, Spinal Cord Research Centre, University of Manitoba, Winnipeg, MB, Canada

**Keywords:** exercise descending command, homeostasis, integrative physiology, locomotion, spinal cord injury

## Abstract

Although the autonomic sympathetic system is activated in parallel with locomotion, the underlying neural mechanisms mediating this coordination are not completely understood. Descending exercise or “central command” signals from hypothalamic and brainstem regions are thought to activate thoracic spinal sympathetic neurons in parallel with descending locomotor commands. In turn, subsets of thoracic sympathetic preganglionic neurons (SPNs) increase activity in a constellation of tissues and organs that provide homeostatic and metabolic support during movement and exercise. It is known that ascending drive from lumbar locomotor networks is mediated in part via propriospinal neurons that can also activate and coordinate autonomic systems. However, the extent to which this ascending drive is distributed to SPNs within thoracic regions is unknown. To investigate this, we applied neurochemicals to elicit whole-cord or lumbar-evoked locomotor activity in an *in vitro* spinal cord preparation, simultaneously recording lumbar ventral root (VR) activity and changes in normalized calcium fluorescence (Ca-RI) of pre-labelled SPNs in thoracic segments. Using whole-bath drug application SPN responses appeared unimodal, such that SPN Ca-RI was increased in rostral (T4-FT7) compared to caudal (T8-T11) segments during tonic activity. During rhythmic activity in either whole or split-bath configuration, and during tonic activity in split-bath configuration, SPN responses appeared trimodal, such that SPN Ca-RI was increased in mid-thoracic segments (T6-7) and reduced at more rostral (T4-5) and caudal (T8-9) levels. In both approaches, the greatest increases in SPNs Ca-RI during rhythmic activity were at T6-7, and most decreased at caudal segments (T8-T11). Together, these findings reveal a strong ascending lumbar to thoracic integrating communication pathway, which may represent a key feature of spinal neural network function normally. Such communication pathways should be further investigated for targeted autonomic function(s) activation and therapeutic benefit after spinal cord injury.

## Introduction

Locomotion and exercise are fundamental behaviors involving sustained rhythmic contractions of muscles, at varying intensities, depending upon the mode of movement and groups of muscles used. It is well established that the generation of a locomotor pattern for overground movement is an intrinsic property of the spinal cord, with important contributions from afferent and descending supraspinal inputs [reviewed in Stuart and Hultborn (2008)]. Descending command signals activate spinal neural circuitry which converts tonic descending drive command signals into rhythmic and well-coordinated locomotor activity, in spinal locomotor or central pattern generator networks (i.e., CPGs) (Shik et al., 1969; Jordan et al., 1979; Grillner, 1981; Whelan, 1996; Jordan et al., 2008).

Electrical stimulation of the mesencephalic locomotor region (MLR) to induce locomotion also causes concomitant increases in blood pressure (Eldridge et al., 1985; Bedford et al., 1992), in part mediated by descending input to spinal sympathetic networks via vasomotor commands from the rostro-ventral lateral medulla (RVLM) (Loewy and Spyer, 1990; Schreihofer and Sved, 2011). Indeed, the RVLM is a key integration site that regulates homeostatic and metabolic functions integral for maintaining whole body homeostasis (i.e., blood pressure, temperature, respiration and heart rate regulation) and for regulating availability of circulating glucose and fatty acids by regulating glucose counterregulatory responses and lipolysis from adipose tissue stores, respectively (Blessing et al., 1999; Morrison et al., 1999; Bartness et al., 2010a, 2010b, 2014; Verberne et al., 2014). However, the neural mechanisms underlying the integrated activation of locomotor and sympathetic autonomic systems are only recently starting to be understood. For example, a monosynaptic glutamatergic excitatory projection from neurons in the MLR to RVLM neurons was recently identified, and optical stimulation of these MLR neurons increased mean arterial pressure and elicited lumbar motor activity in decerebrate rats, and increased mean arterial pressure and speed of ongoing locomotion in freely moving rats, suggesting locomotor and autonomic sympathetic systems were functionally integrated (Koba et al., 2022). Further, chemogenetic and optogenetic activation of serotonergic neurons in the parapyramidal region first increases blood pressure and then hindlimb locomotor activity in adult decerebrate rats (Armstrong et al., 2017; Armstrong, 2024), suggesting integration and simultaneous activation between locomotor and sympathetic autonomic systems occurs at multiple levels within the brainstem.

At the level of the spinal cord, clinical observations that spinal electrical stimulation in lumbar locomotor regions can improve or normalize a subset of movement and exercise-related metabolic and homeostatic functions in motor complete spinal cord injury [reviewed in Flett et al. (2022)], yet less is known about whether and how spinal locomotor and sympathetic systems are integrated. In C1-spinalized L-Dopa cats, Schomburg et al. (2003) demonstrated that rhythmic cardiac and cervical sympathetic nerve activity occurred in 2/3 of preparations in which stable alternating locomotor activity occurred. The frequency of sympathetic nerve discharge was entrained with the hindlimb locomotor activity. Although membrane oscillations in SPNs were rarely seen during excitatory amino acid receptor agonist N-methyl-D-aspartate (NMDA) and serotonin (5HT)-induced rhythmic ~0.4 Hz VR activity *in vitro* (2/18 rats), oscillations were observed in SPNs during very slow irregular rhythmic VR activity (0.03–0.06 Hz) induced by the non-selective cholinergic agonist oxotremorine (Sourioux et al., 2018). Recently, we demonstrated that a class of genetically identified, locomotor-related neurons (V3 interneurons (INs)) in the lumbar region provide direct excitatory glutamatergic synaptic projections onto thoracic spinal preganglionic sympathetic neurons (SPNs), and that optical stimulation of lumbar V3 INs generates action potentials in SPNs (Chacon et al., 2023). Thus, at least one class of spinal locomotor-related neurons contribute to excitation of thoracic SPNs, demonstrating a neural substrate/mechanism of intraspinal ascending locomotor-sympathetic system integration. Taken together, these findings indicate the spinal cord can generate rhythmic and coordinated hindlimb locomotor discharge integrated with sympathetic nerve activity in the absence of any direct descending input from supraspinal centres, although contributing neural mechanisms and pathways remain to be identified.

Although SPN activity in response to application of glutamate, 5HT or dopamine (DA) receptor agonists has been characterized in slice or spinal preparations (Spanswick and Logan, 1990; Pickering et al., 1994; Gladwell and Coote, 1999a; Zimmerman et al., 2012), SPN population responses during rhythmic locomotor activity have not been described. Additionally, SPN responses to the locomotor activity-inducing combination of these neurotransmitters has not been described or characterized by segmental level. Thus, the present work examines the effect(s) of tonic and rhythmic locomotor activity on SPN responses at different thoracic rostro-caudal levels (T3/4 through T11) in response to either whole-bath or lumbar-bath application of locomotor inducing neurochemicals. The results provide physiological evidence that ascending propriospinal pathways contribute to lumbar locomotor-mediated activation of sympathetic neurons in particular thoracic segments, while decreasing activation of SPNs at other thoracic levels. Preliminary results were presented previously in abstract form (Nwachukwu et al., 2022; Dominguez-Rodriguez et al., 2024).

## Methods

### Animals

Experimental protocols used complied with the guidelines set by the Canadian Council on Animal Care and were approved by the University of Manitoba animal ethics committee. In our initial set of experiments, postnatal day 0 (P0) to P5 pups from our in-house C57Bl/6 colony were used. In all subsequent experiments, B6.129S-Chat^tm1(cre)Lowl^/MwarJ (Jax strain # 031661) were crossed with B6J. Cg-Gt(ROSA)26Sor^tm96(CAG-GCAMP6s)Hze^/MwarJ (Jax Strain # 028866) to generate B6.129S-Chattm1(cre0Lowl/Gt(ROSA)26Sortm96(CAG-GCamp6s)Hze/Mwar) mice (referred to ChatCre/Gcamp6s). Pups (P0-P5) from the ChatCre/Gcamp6s endogenously express the genetically encoded calcium indicator (GCamp6s) in cholinergic SPNs and motoneurons (MNs) and were used for experimentation.

### *In vitro* electrophysiology

#### Whole spinal cord preparation with obliquely cut surface

Spinal cords were dissected from either C57BL/6 (*n* = 26) or ChAT-Cre/GCaMP6S (*n* = 30) mice, as previously described (Chopek et al., 2018; Chacon et al., 2023). Briefly, animals were anesthetized with isoflurane (Fresenius Kabi Canada Ltd., Ontario, Canada) decapitated at the medulla-spinal cord junction, eviscerated, and spinal cords dissected out in ice-cold (~4 °C) dissecting artificial cerebrospinal fluid (aCSF), composed of (mM): KCl (3.5), NaHCO_3_ (35), KH_2_PO_4_ (1.2), MgSO_4_ (1.3), CaCl_2_ (1.2), glucose (10), sucrose (212.5), and MgCl_2_ (2.2). The dissecting aCSF solution was equilibrated to pH 7.4 and continuously oxygenated (95% O_2_; 5% CO_2_). Spinal cords were then mounted on an agar block and fixed in place with acrylic glue for sectioning using a vibratome (Leica VT1200S, Leica Biosystems, Ontario, Canada). The level of the oblique slice was recorded for each preparation and used in *post hoc* analysis to determine if SPN calcium imaging responses varied at different thoracic segments. The exposed portion of the spinal cord was then glued to a sylgard-coated (Sylgard, Dow Corning, Michigan, USA) recording chamber designed and 3D-printed in-house. Specifically, the method is a modification of that described by (Rancic et al., 2019), in which the obliquely cut surface of the spinal cord was placed on a sylgard “ramp” (~30^o^) to enable simultaneous visualization SPNs under fluorescence while maintaining physical continuity with the lumbar spinal cord for generating lumbar locomotor activity, as monitored by ventral root (VR) recordings. All recordings were performed in oxygenated room-temperature aCSF composed of (mM): NaCl (111.0), KCl (3.085), D-glucose (10.99), NaHCO_3_ (25.0), MgSO_4_.7^. H^_2_O (0.31), CaCl_2_ (2.52), KH_2_PO_4_ (1.1); pH 7.4.

#### Whole cord and lumbar-evoked induction of tonic and rhythmic motor activity

In our first series of experiments, whole-bath application of neurochemicals was used to elicit locomotor activity in C57Bl/6 mice (*n* = 26) with varying combinations of 5HT, NMDA and DA, in the following concentrations: 5HT (Sigma-Aldrich Co., MO, USA; 10–50 μM), NMDA (Sigma-Aldrich; 2–10 μM), and DA (Sigma-Aldrich; 50–100 μM). In some experiments di-hydro-kainic acid (DHK, Tocris Bioscience, Bristol, UK; 150–400 μM, *n* = 16 trials in 14 mice), and bicuculline (BIC, Sigma-Aldrich; 10–40 μM, *n* = 4 trials in 4 mice) were also applied to induce rhythmic activity. Neurochemical concentrations refer to final bath concentrations.

Using ChatCre/GCamp6s mice in another series of experiments (*n* = 15), a split-bath preparation was used to isolate the lumbar spinal cord from the thoracic spinal cord with a 3D-printed 2-chamber bath sealed at the chamber barrier and spinal cord contact edges with petroleum jelly (Cowley and Schmidt, 1997). A dual-perfusion system was used to apply the locomotor cocktail (20 μM 5HT, 5 μM NMDA and 5 μM DA in P0-2 and 30 μM 5HT, 5 μM NMDA and 10 μM DA in P3-5 mice) exclusively to the lumbar region, while the thoracic region was perfused with oxygenated recording aCSF. Drugs were then applied only to the lumbar spinal region to evoke locomotor activity which allowed us to record SPN activity in the absence of direct effects of the neurochemicals on SPNs. Red food coloring dye placed in the lumbar chamber following experimentation was used to confirm integrity of seal and that drug application was confined to the caudal bath segment.

#### Lumbar ventral root monitoring and recording

Lumbar VR recordings were obtained using glass suction electrodes (120–140 μm inner diameter). We classified tonic activity as a sustained increase in VR discharge compared to baseline whereas rhythmic locomotor activity was defined by VR bursting between left and right L2 or L5, or between ipsilateral L2 and L5 VRs since L2 corresponds with flexor, and L5 with extensor phases of the step cycle, respectively (Cowley and Schmidt, 1994; Kiehn and Kjaerulff, 1996). For technical reasons and space constraints, we targeted recording from 2 to 3 VRs and were not always able to record from L2 and/or L5 VRs and elected to use other VRs (L3, L4). As described previously, rhythmic discharge in these VRs does not always display rhythmic left–right and/or ipsilateral presumed flexor-extensor alternation (Cowley and Schmidt, 1994) and we therefore refer to this type of activity in one or more VRs as rhythmic motor activity. VR signals were band-pass filtered at 30–3,000 Hz, amplified (custom-made SCRC amplifier), digitized and acquired in gap-free mode at 2 kHz with a CED Power 1,401 AD board, and displayed with Signal 7.6 Software (Cambridge Electronic Devices, Cambridge, UK). All recordings were performed at room temperature (~22 °C). A TTL pulse from the camera to the electrophysiological data capture system (Signal 7.6, Cambridge electronic devices, Cambridge, UK) synchronized calcium imaging with VR recordings.

### Calcium imaging

#### Retrograde labeling of thoracic SPNs

**Figure 1:**
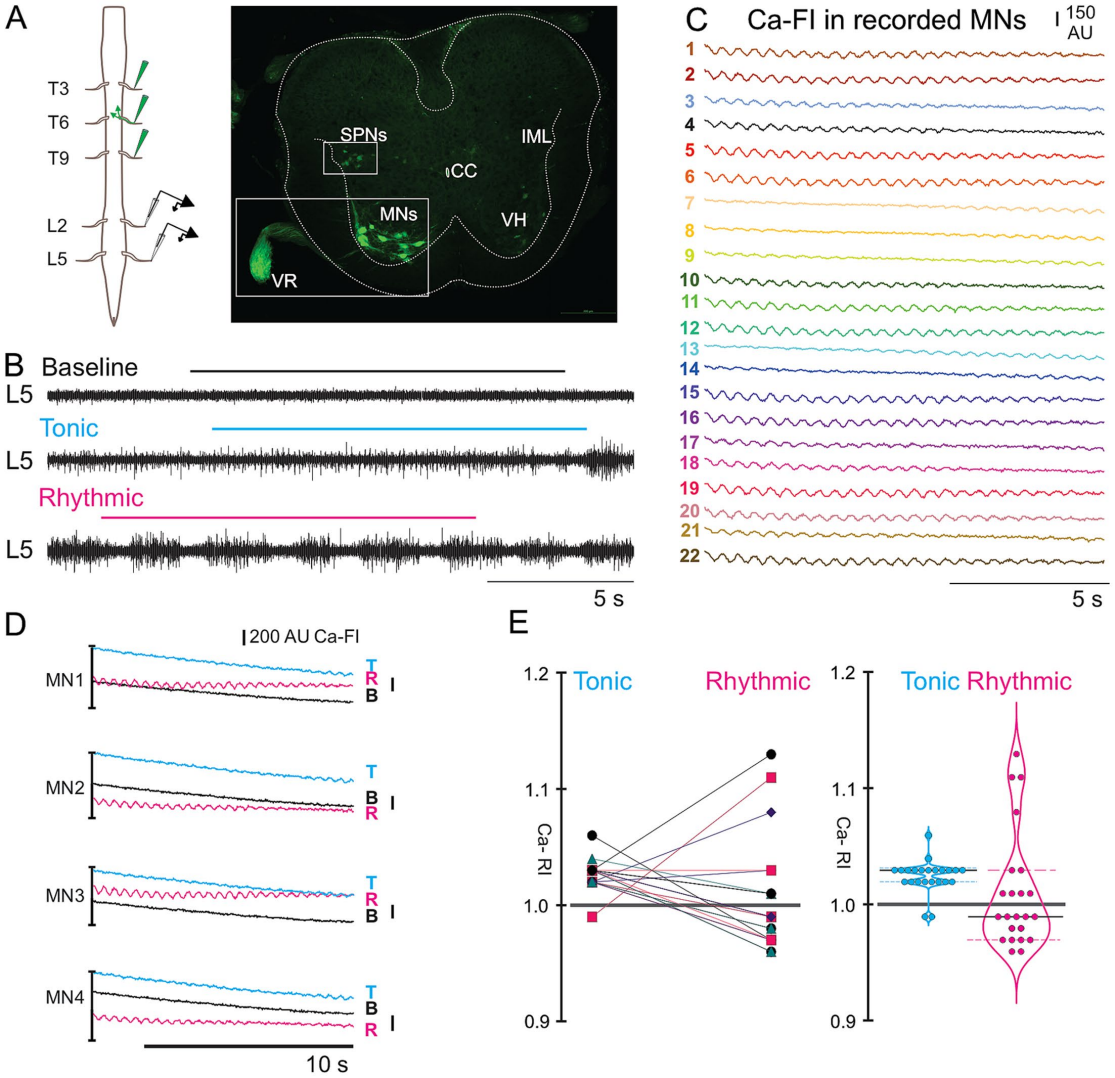
Calcium responses in thoracic motoneurons (MNs) during tonic and rhythmic ventral root (VR) activity. **(A)** *Schematic* of the retrograde dye labeling procedure and lumbar VR recording set-up (*left*). Transverse section of thoracic spinal cord with retrogradely labeled MNs and SPNs (*right*). **(B)** VR recordings at baseline, and during tonic and rhythmic activity. **(C)** Calcium imaging responses of 22 motoneurons at T10–11 during rhythmic activity induced by 20 μM 5-HT, 5 μM NMDA, and 50 μM DA. **(D)** Ca-FI traces from four representative MNs (MNs 1–4 in C), shown during baseline, tonic, and rhythmic VR activity. Ca-FI increased during tonic activity with some transitioning to rhythmic oscillations. Note that Ca-Fl intensity levels were higher during tonic versus rhythmic activity for each of these 4 MNs and that rhythmic oscillations were clearly evident during rhythmic VR activity. Calcium intensity is shown in arbitrary units (AU, scale bar top right). **(E)** Individual Ca-RI (*left),* and violin plots (*right*) for all 22 MNs during tonic and rhythmic VR activity. Most MNs showed an initial increase in Ca-RI during tonic activity compared to normalized baseline, which may further increase during rhythmic activity. Violin plots summarizing Ca-RI values indicated that while tonic activity led to a general increase in Ca-RI for most MNs, during rhythmic activity, a subset of MNs exhibited a decrease in Ca-RI. For this and all subsequent figures, the thick black line within each violin plot is the median of all Ca-RI values and the thinner dashed lines indicate the 1st and 3rd quartiles. Tonic VR activity median = 1.03, rhythmic VR activity median = 0.99. For this and all other figures, baseline is indicated in black, tonic activity with cyan and rhythmic activity with magenta. Ai and Aii refers to left and right.

After dissection, spinal cords from neonatal C57BL/6 mice (P0-P5) were transferred (ventral side up) into a chamber with oxygenated room-temperature aCSF. SPNs and somatic MNs from different thoracic segments (T4-T12) were retrogradely labeled by applying calcium dye (Ca-green conjugated dextran amine, CGDA; 3,000 MW, Life Technologies Corp., OR USA) to the cut ends of thoracic VRs (Th-VRs, see Figure 1Ai). CGDA crystals were diluted with aCSF into 20% stock solution (25 μL of aCSF / 5 mg of CGDA crystals). A small volume (4-6 μL) of the diluted calcium dye was applied to thoracic VRs using suction glass microelectrodes (100-120 μm inner diameter). Thoracic VRs were cut immediately before suctioning to increase dye uptake and sectioned close to their exit from the spinal cord to minimize labelling time. Thoracic VRs were selected based on viability and length. Retrograde labeling of corresponding thoracic MNs and SPNs continued in the dark at room temperature (~22 °C) for at least 3 h (Szokol et al., 2008).

#### Thoracic SPNs visualization

Labeled thoracic SPNs (or MNs), either by retrograde calcium dye or by genetically encoded calcium indicator GCaMP6s, were visualized in obliquely cut cords at different thoracic levels (T3-T12), as described above. The thoracic surface was visualized first using 5× wide objective lens (Axio Examiner. Z1 microscope; Göttingen, Germany) for a boarder view, identifying ventral and dorsal horns and the central canal. Then, under fluorescence with the 20× wide objective, clusters of labeled SPNs were identified based on their lateral location and position relative to the central canal, where the intermediolateral cell column (IML) is located. MNs pools were clearly distinguishable from SPNs based on their size and ventral location in lamina IX (see Figure 1Aii).

#### Optical recording of calcium responses

Thoracic SPNs were imaged using a 20× wide aperture (1.2 nA) water-immersion objective lens on an upright epi-fluorescence Zeiss Axio Examiner microscope. Fluorescence intensity changes were recorded using Prime BSI Scientific CMOS camera (Photometrics, BC, Canada) and SlideBook 6.0 software (Intelligent Imaging Innovations, Denver, CO, USA, RRID: SCR_014300). Image series were captured at sampling frequencies between 4.5–7.5 Hz, and occasionally at 40 Hz. Regions of interest (ROIs) were drawn around the somas of labeled thoracic SPNs in the IML or MNs in the ventral horn to record fluorescence intensity at baseline (before drug-induced locomotion) and during tonic and/or rhythmic VR activity. We set a background fluorescence region as an area within the imaged field far from labelled thoracic SPNs to normalize for changes in global fluorescence changes over time during each experiment.

### Data analysis

#### Post-hoc normalization and analysis procedures

For all analyses, calcium fluorescence intensities (Ca-FI) for each SPN were exported from Slidebook software into Microsoft Excel. To compare Ca-FI at baseline and during tonic and rhythmic VR-activity for individual SPNs, each CA-FI was normalized to the background fluorescence region in each condition. This allowed us to calculate the relative intensity (Ca-RI) of normalized fluorescence intensity for each ROI during tonic and rhythmic activity and express it as a percentage of baseline fluorescence (Ratliff et al., 2023). For some animals, files were converted into text files and imported into either Analysis (in-house SCRC software) or Signal 7.6 Software for subsequent oscillatory calcium event visualization (plotting) and analysis. For MNs, oscillations in Ca-RI within each Slidebook capture window were robust, consistent and easily visually identifiable, particularly when sampling at 40 Hz. Data were exported as text files into GraphPad Prism Software, San Diego, California, USA, for graphing and statistical analysis. A 3-way ANOVA, with Tukey multiple comparisons correction was used to assess Ca-RI (mean, SD, n) using thoracic level (T4-T5, T6-T7, T8-T9), bath configuration (split, whole) and lumbar VR condition (tonic, rhythmic) as the three nominative variables. Averages are expressed as means ± standard deviations, and ranges denoted in square parentheses [], *p*-values are multiplicity-adjusted as appropriate.

## Results

### Ca-RI responses observed in MNs during tonic and rhythmic locomotor-like activity during whole bath-application of neurochemicals

Initially we recorded activity in 56 CDGA retrogradely labelled thoracic MNs (6 preparations, T7-T10, see dye application and VR recording set up Figure 1A) and from 19 thoracic MNs that expressed GCaMP6s indicator (4 preparations, T4-5) to test our methodology in comparison to previous studies. As expected, at baseline, CDGA-labelled MNs did not demonstrate oscillatory transients in Ca-FI. Also as expected, we observed regular oscillations in Ca-FI recorded at T10-11 in CDGA-labelled thoracic MNs during rhythmic VR activity (Figures 1B–D), consistent with others’ findings of rhythmic oscillations in motoneurons and locomotor-related spinal interneurons during *in vitro* locomotor activity (Jean-Xavier and Perreault, 2018; Rancic et al., 2020). During rhythmic VR activity, many of the 22 MNs displayed rhythmic oscillations throughout each 12-s record (e.g., Figure 1C, MNs 1–2, 5–6), whereas oscillations in other MNs appeared episodically, occurring at the beginning or end of each record (e.g., Figure 1C, MNs 7–9, 13–14). During tonic VR activity that typically precedes the appearance of rhythmic VR activity, most MNs showed increased Ca-FI (Figure 1D, cyan), with some also displaying rhythmic oscillations in Ca-FI. Relative Ca-FI was lower during rhythmic activity in these 4 MNs (Figure 1D, magenta) when compared to during tonic discharge.

Similarly, most of the 22 MNs examined displayed an increase in Ca-RI during tonic VR activity (Figures 1D,E, see tonic in before/after and violin plots), likely related to increased resting membrane potentials of MNs observed during tonic VR activity (Krawitz et al., 2001; MacDonell et al., 2015; Jean-Xavier and Perreault, 2018). During rhythmic VR activity, responses were more variable, with increased Ca-RI in 6/22 (27%), Ca-RI remaining relatively similar to baseline (*Δ* ≤ ± 3%) in 9/22 (41%) and decreased Ca-RI in 8/22 (32%) MNs (Figure 1D, right). The regular MN Ca-RI oscillations observed during rhythmic VR activity likely reflects underlying locomotor drive potentials (LDPs) observed during fictive locomotion (Jordan, 1983; Hochman and Schmidt, 1998). The MN Ca-RI intensity changes we observed were similar to those observed in thoracic MNs in response to brainstem stimulation (Szokol et al., 2008), and showed rhythmic oscillations similar to those observed in MNs and presumed locomotor-related INs during rhythmic lumbar locomotor activity (O'Donovan et al., 2005; Jean-Xavier and Perreault, 2018; Rancic et al., 2020).

### SPN responses during whole-bath application of neurochemicals

#### Thoracic SPN ca-RI responses during lumbar VR locomotor activity induced by whole-cord drug application

**Figure 2:**
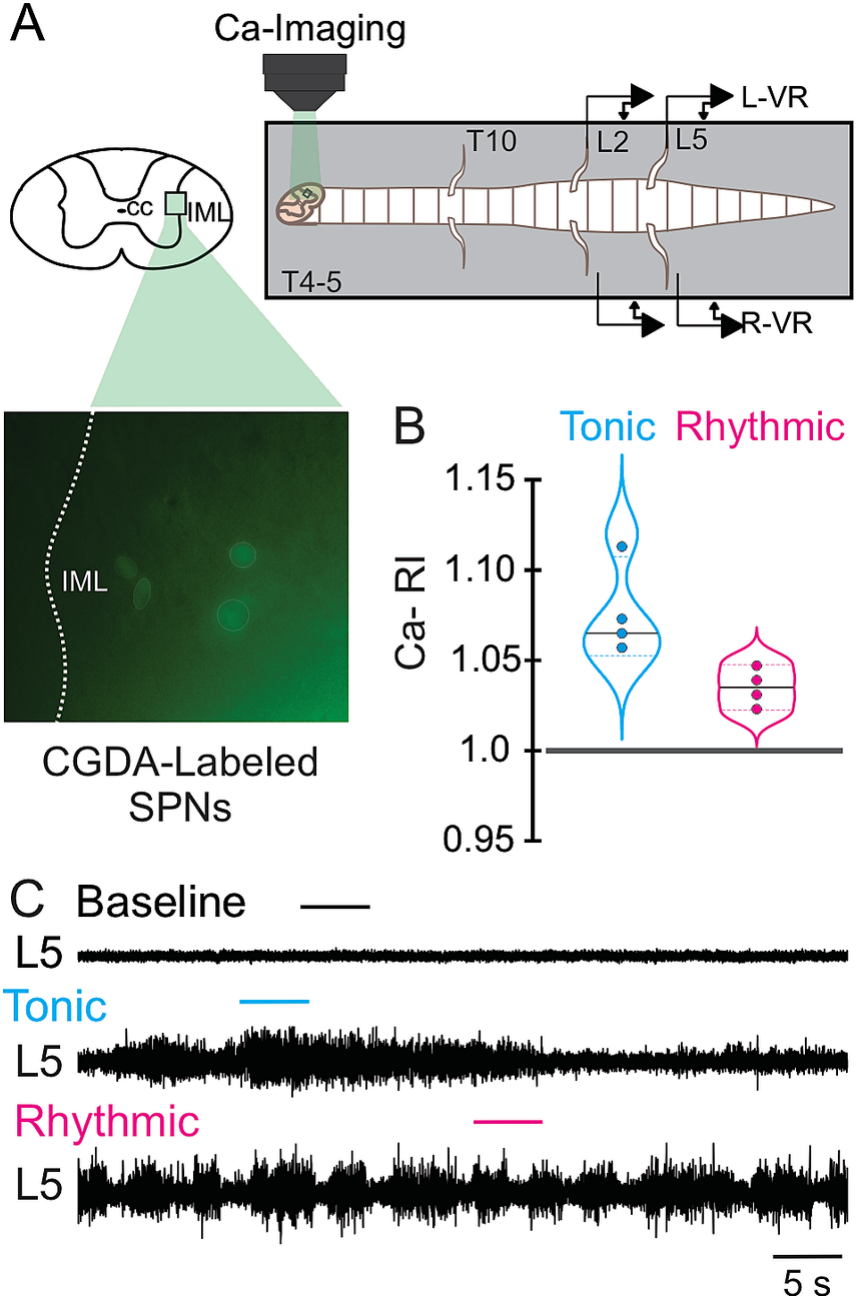
Calcium responses of thoracic SPNs during VR activity induced by whole-bath application of 5-HT, NMDA, and DA. **(A)** Schematic showing transverse section of spinal cord with rectangle highlighting IML region of interest (*top left*); image below shows masks over cell bodies of four CGDA-retrogradely labeled thoracic SPNs at T6–7 (*bottom left*); and calcium imaging set up with lumbar VR recordings is shown in accompanying schematic (*top right*). **(B)** Violin plots show changes in calcium relative intensity (Ca-RI) corresponding to VR activity in **(C)**. Baseline, tonic, and rhythmic VR activity shown for a single L5 VR. Violin plot Ca-RI values are normalized to baseline background fluorescence (marked by line at 1.0). Recordings were obtained during 12 s calcium imaging episodes sampled at 7.8 Hz. Note that Ca-RI increased more during tonic (median = 1.065) versus rhythmic (median 1.035) activity.

**Figure 3:**
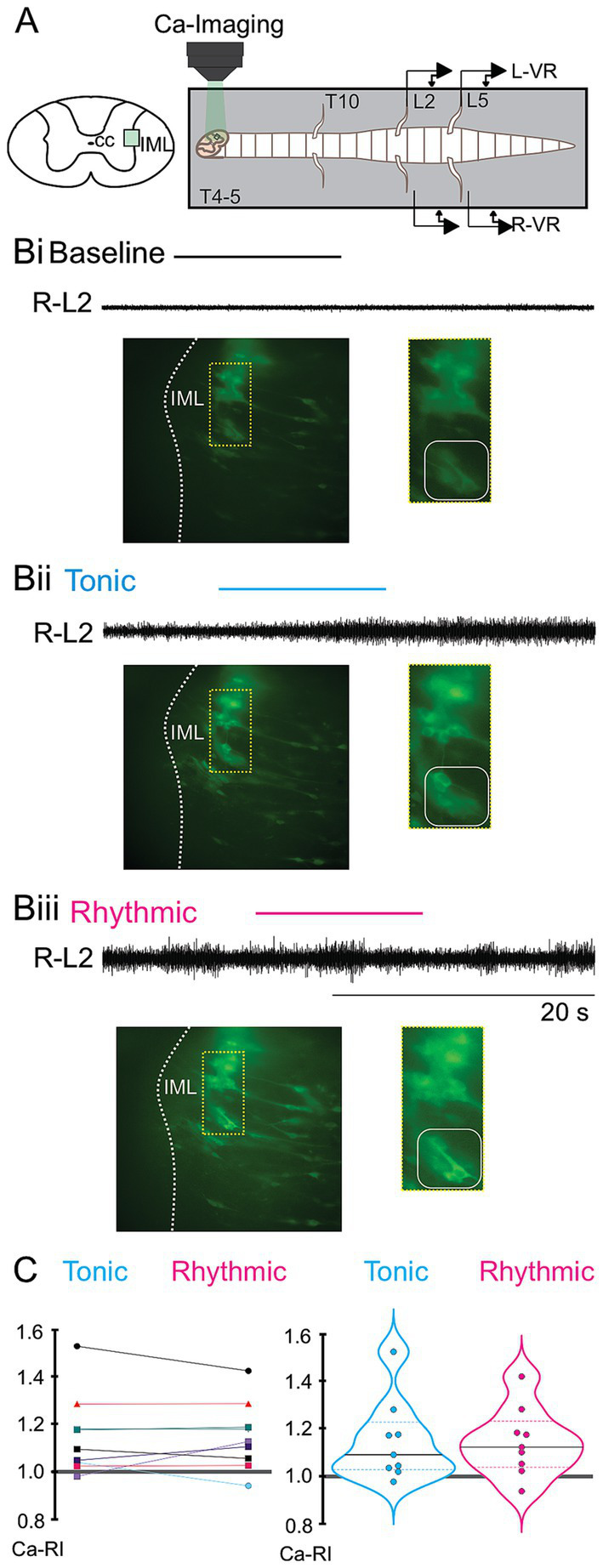
Increased recruitment of thoracic SPNs during tonic and rhythmic VR activity. **(A)** Schematic showing transverse section of spinal cord with rectangle highlighting IML region of interest (*left*) and experimental set-up for SPN imaging at T4–5 within the whole-bath configuration for applying locomotor-inducing neurochemicals to the entire spinal preparation. **(B)** Representative calcium images of SPNs at the T4–5 level in a GCaMP6s P2 mouse with VR recordings at baseline **(i)**, and during tonic **(ii)** and rhythmic **(iii)** VR activity induced by 20 μM 5-HT and 5 μM NMDA. Images below each electroneurogram show clusters of SPNs with images captured during baseline, tonic, and rhythmic activity. As highlighted by the white box in the magnified images on the right, two SPNs exhibit visibly progressive increases in Ca-Fl during tonic and then rhythmic activity. **(C)** *Left panel:* Individual Ca-RI values for the 9 recorded SPNs during tonic and rhythmic VR activity. Most SPNs (8/9) had increased Ca-RI compared to the normalized baseline (shown as the solid black line at 1.0) during tonic activity. Similarly, most SPNs (8/9) had increased Ca-RI during rhythmic VR activity compared to normalized baseline (note a different SPN showed reduced Ca-RI). *Right panel:* Violin plots summarizing Ca-RI values for the recorded SPNs during tonic and rhythmic activity with tonic VR activity median = 1.09, and rhythmic VR activity median = 1.10.

**Figure 4:**
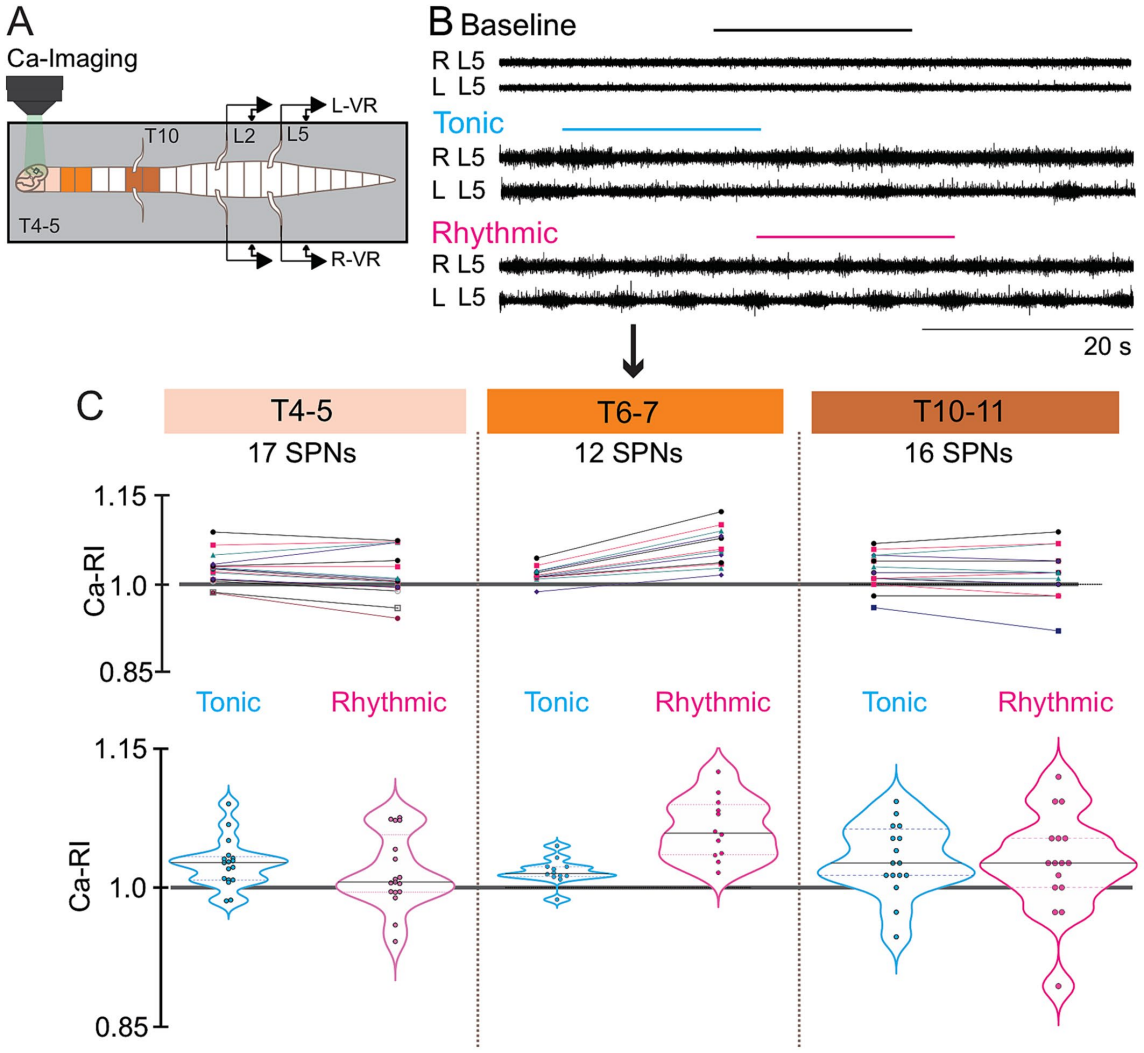
Thoracic SPN Ca-RI responses recorded from a variety of thoracic segmental levels in response to whole cord drug application. **(A)** Experimental set up for the whole-bath configuration, as in Figure 3. **(B)** Electroneurogram recordings during baseline, tonic and rhythmic L5 VR activity with timing of simultaneous imaging of SPNs at T6-7 shown by colored bars above each trace. **(C)** Ca-RI responses for 3 different experiments recorded T4-5, T6-7, and T10-11 (colors reference segments shown in schematic in A). Upper panels show individual Ca-RI responses during tonic and rhythmic VR activity, normalized to baseline, with corresponding violin plots shown in lower panels. The majority of SPNs at T4-5 in this experiment (*left*) showed increased Ca-RI during tonic VR activity whereas fewer (~ half) had increased Ca-RI during rhythmic VR activity. Violin plots indicate a slight increase in Ca-RI during tonic VR activity (median = 1.03), with SPN responses during rhythmic VR activity more variable, with 5 increasing, 2 decreasing, and most remaining similar to baseline (median = 1.01). At T6-7 (*middle*), SPNs showed greater increases in Ca-RI during rhythmic (median = 1.06) compared to tonic VR activity (median = 1.01). Although overall there was a small increase in median Ca-RI during both tonic (1.02) and rhythmic (1.01) VR activity in SPNs recorded at T10-11, the connecting line plots show the mixed responses of individual SPNs.

We used two approaches to characterize thoracic SPNs responses during lumbar locomotor activity. In the first, we perfused the entire spinal cord with neurochemicals to induce fictive locomotion while recording SPN population calcium responses at a variety of rostrocaudal levels (T4 to T11), monitoring SPN activity levels at baseline, and during tonic and rhythmic activity. In our second approach we limited neurochemical application to T13 and caudal to remove the direct effect(s) of neurochemical application on SPNs while maintaining the ability to generate hindlimb locomotor activity (Cazalets et al., 1995; Kjaerulff and Kiehn, 1996; Cowley and Schmidt, 1997; Kremer and Lev-Tov, 1997; Cowley et al., 2009), see below. Calcium image recordings were performed from 313 CGDA labelled thoracic SPNs (25 preparations) and 192 thoracic SPNs in our ChatCre/Gcamp6S mouse (15 preparations) in our whole-bath configuration experiments. SPNs recorded from segments T4 through T11 displayed a variety of responses at baseline, and during tonic and rhythmic activity (see schematics in Figures 2A–4A). For example, the 4 CGDA-labeled SPNs recorded using retrograde labeling from T4-5 (Figure 2) all showed increased Ca-RI that was greater during tonic (cyan) compared to during rhythmic (magenta) VR activity (Figures 2B,C), as we had observed in MNs (compare Figure 2B to Figure 1E). As noted above, here and elsewhere, only changes > ± 3% were classified as increased or decreased Ca-RI. Whenever possible, we recorded tonic activity to determine if SPNs displayed changes in Ca-RI similar to MNs before onset of regular and rhythmic VR discharge developed.

Our initial focus was to determine if we would see rhythmic oscillations in SPNs during rhythmic lumbar locomotor activity, similar to that observed in our recorded thoracic MNs. However, we did not observe any consistent rhythmic oscillatory activity in SPNs. The absence of any observed rhythmic activity may be due to a variety of factors, including much smaller SPN size and relative fluorescence compared to MNs, low intrinsic probability and/or frequency of SPN oscillations or rhythmic bursting (<1 Hz), and/or imaging rates too low (4–8 Hz) to detect higher frequency SPN oscillations (>10 Hz) or that SPNs show only tonic/continuous Ca^2+^ fluorescence increases or decreases during lumbar motor activity. Perreault and colleagues detected rhythmic oscillations SPNs in response to vestibular stimulation, but only when recording at 100 Hz whereas only tonic intensity changes were observed when recording at their typical 4 Hz imaging rate (Kasumacic et al., 2012). Additionally, Sourioux et al. (2018) reported SPN membrane oscillations were only rarely seen during lumbar VR motor rhythms induced by 5HT and NMDA. Thus we focused on examining changes in Ca-RI at different thoracic segmental levels during tonic and rhythmic locomotor activity.

In the preparation shown in Figure 3, recordings from a ChatCre/Gcamp6S mouse were obtained at T4/5 before and after whole-bath application of 20 μM 5HT and 5 μM NMDA (schematic of set up in Figure 3A). Some Ca-RI increases were readily evident upon visual inspection of the calcium images (Figure 3B images with magnified insets) whereas others required normalization to averaged baseline values and analyses to illustrate changes in Ca-RI (Figure 3C). The images in Figures 3Bi–Biii show that most of the visualized thoracic SPNs progressively increased their Ca-FI during tonic and then rhythmic activity, with two SPNs highlighted in the smallest insets. Overall, most SPNs in this preparation displayed increased Ca-RI during tonic and rhythmic activity (Figure 3C), with only one SPN showing decreased Ca-RI compared to baseline during tonic (Figure 3C, left before/after plot, purple line/dot) and another SPN with decreased Ca-RI compared to baseline during rhythmic activity (Figure 3C, left cyan line/dot).

#### SPNs exhibit distinct patterns of ca-RI responses at different thoracic segmental levels and differed during tonic and rhythmic lumbar locomotor-like activity

During whole cord drug application, we observed differences in the pattern of Ca-RI responses during tonic compared to rhythmic VR activity and at different spinal segments (Figure 4, schematic in A shows segments colour-coded to correspond with examples shown in C). The VR activity in Figure 4B was a preparation in which SPNs were recorded at T6-7 (arrow, middle panel in Figure 4C), in which Ca-RI was increased in 11/12 SPNs during tonic (Figure 4C middle panel, left) and in all during rhythmic (Figure 4C middle panel, right) VR activity. As can be seen by the examples shown for SPNs recorded from either T4-5 (Figure 4C, left panel) or T10-11 (Figure 4C, right panel), responses were mixed at these levels such that a larger portion of SPNs had decreased Ca-RI during both tonic and rhythmic activity (compare before/after and violin plots in Figure 4C).

In summary for the whole cord configuration (left), we recorded 164 SPNs at the T4-5 level during tonic activity (11 preparations, 12 trials), and Ca-RI increased in 82/164 (50%), was unchanged in 57/164 (35%), and decreased in 25/164 (15%). Of the 153 thoracic SPNs recorded at T4-5 during rhythmic VR activity (9 preparation, 12 trials), Ca-RI increased in 51/153 (33%), was unchanged in 53/153 (35%), and decreased in 49/153 (32%). Thus, while 50% of SPNs in T4-5 segments showed increased Ca-RI during tonic VR activity, fewer (33%) increased Ca-RI during rhythmic VR activity.

At the T6-7 level in the whole bath configuration, we recorded 47 SPNs during tonic VR activity (5 preparation, 6 trials), and Ca-RI increased in 23/47 (49%), was unchanged in 23/47 (49%), and decreased in 1/47 (2%). Of the 39 thoracic SPNs recorded at T6-7 during rhythmic VR activity, Ca-RI increased in the majority of SPNs (87%, 34/39) and was unchanged in 5/39 (13%). Thus, a greater proportion of SPNs at T6-7 displayed increased Ca-RI during rhythmic (87%) compared to during tonic (49%) activity with whole bath drug application.

Only a small proportion of recorded SPNs displayed increased Ca-RI at caudal thoracic levels in the whole bath configuration. During tonic activity at T8-9, only 7% of SPNs (6/82) had increased Ca-RI (8 preparations, 9 trials) and 17% (9/114) at T10-11 (12 preparations, 14 trials). Similarly, low numbers of SPNs displayed increased Ca-RI during rhythmic activity at these levels. Specifically, 11/93 (12%) SPNs at T8-9 and 20/167 (12%) at T10-11 levels displayed increased Ca-RI during rhythmic activity. Most commonly, SPNs at T8-T11 showed no change in Ca-RI during either tonic or rhythmic activity (77% at T8-9 and 74% at T10-11).

### SPN responses during split-bath application of neurochemicals

#### Ca-RI responses in thoracic SPNs during tonic and rhythmic locomotor-like activity induced by selective application of neurochemicals to the lumbar spinal cord

**Figure 5:**
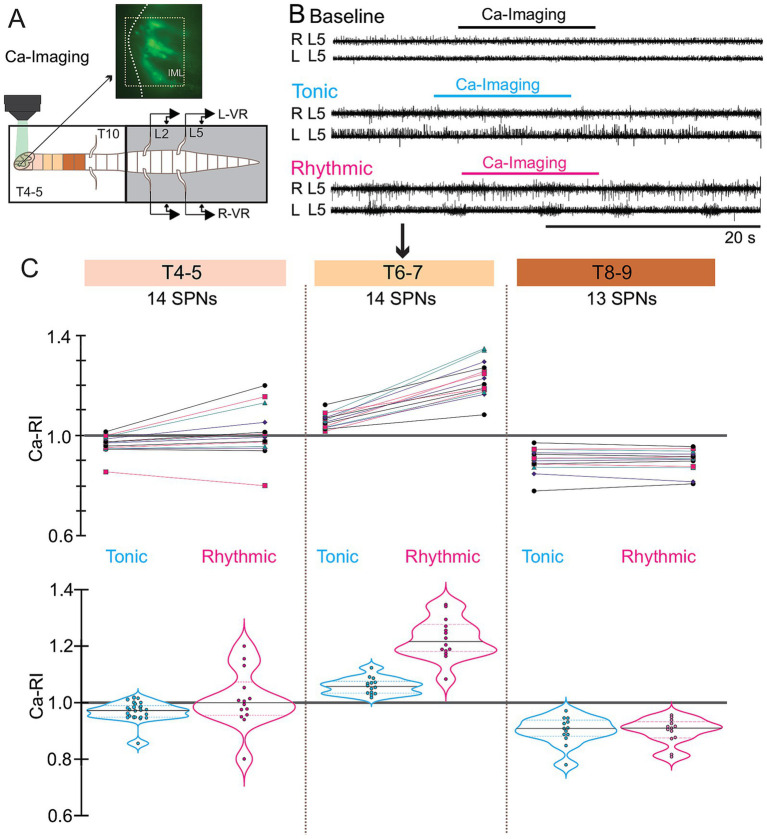
Thoracic SPN Ca-RI responses recorded from a variety of thoracic segmental levels in response to lumbar spinal cord drug application. **(A)** Schematic showing split-bath configuration with barrier to physically separate thoracic and lumbar regions for selective pharmacological activation of lumbar spinal neurons (gray rectangle, *bottom*), and photomicrograph showing SPNs imaged at T6-7 level in a GCaMP6s P4 mouse (*top*). **(B)** Corresponding neurogram recordings during baseline, tonic, and rhythmic L5 VR activity with timing of simultaneous imaging of SPNs at T6-7 shown by coloured bars above each trace. **(C)** Ca-RI responses for 3 different representative experiments recorded at T4-5, T6-7, and T8-9 [colors reference segments shown in schematic in **(A)**]. Upper panels represent the individual Ca-RI responses, with corresponding violin plots shown in lower panels. Most SPNs at T4-5 (left) showed decreased Ca-RI during tonic VR activity, as represented by a median change to 0.95 in the violin plot, whereas about half had increased Ca-RI during rhythmic VR activity with the averaged response near baseline (median = 1.01). SPNs at T6-7 segment (middle), exhibited a larger increase in Ca-RI during rhythmic (median = 1.22) versus tonic (median = 1.06) VR activity. SPNs in T8-9 segments displayed decreased Ca-RIs during both tonic (median – 0.90) and rhythmic (median = 0.89) VR activity.

In our second approach, we removed any potential direct effects of the neurochemicals on SPNs by using a split-bath configuration, in which locomotor-inducing neurochemicals were applied selectively below the T12/13 spinal level. Thus, the split-bath configuration enabled perfusion of neurochemicals to the chamber containing the lumbosacral spinal cord to activate only the lumbar portion of the locomotor CPG, thereby enabling assessment of the influence of ascending propriospinal projections to thoracic SPNs during lumbar-evoked locomotor activity in the absence of any direct effect(s) of the neurochemicals on SPNs or thoracic INs. We recorded calcium responses from 191 thoracic SPNs (15 preparations) from different thoracic segments at baseline and during tonic and rhythmic VR activity (schematic of set-up and sample of Ca image in Figure 5A).

In the split-bath configuration, thoracic SPNs also exhibited distinct patterns of SPN Ca-RI responses during tonic and rhythmic VR activity at different spinal levels (Figures 5B,C). These calcium responses showed some similar trends to those observed during whole-bath application of locomotor-inducing neurochemicals such that SPN Ca-RI was increased at T6-7 levels during tonic and rhythmic activity (Figures 5B,C middle panel), whereas SPN Ca-RI was mainly decreased at caudal thoracic levels (T8-9, Figure 5C right panel) during tonic and rhythmic activity. In contrast to whole bath application, SPN Ca-RI at T4-5 was either decreased or unchanged during tonic activity in the split-bath configuration (compare Figure 5C, left panel to Figure 4C, left panel). SPN responses at T4-5 appeared similar during rhythmic activity in the split and whole bath configurations, such that similar proportions of SPNs showed increased, unchanged or decreased Ca-RI (compare Figure 5C left panel with Figure 4C left panel).

#### SPNs exhibit distinct patterns of ca-RI responses at different thoracic segmental levels that differed during tonic versus rhythmic lumbar locomotor-like activity

**Figure 6:**
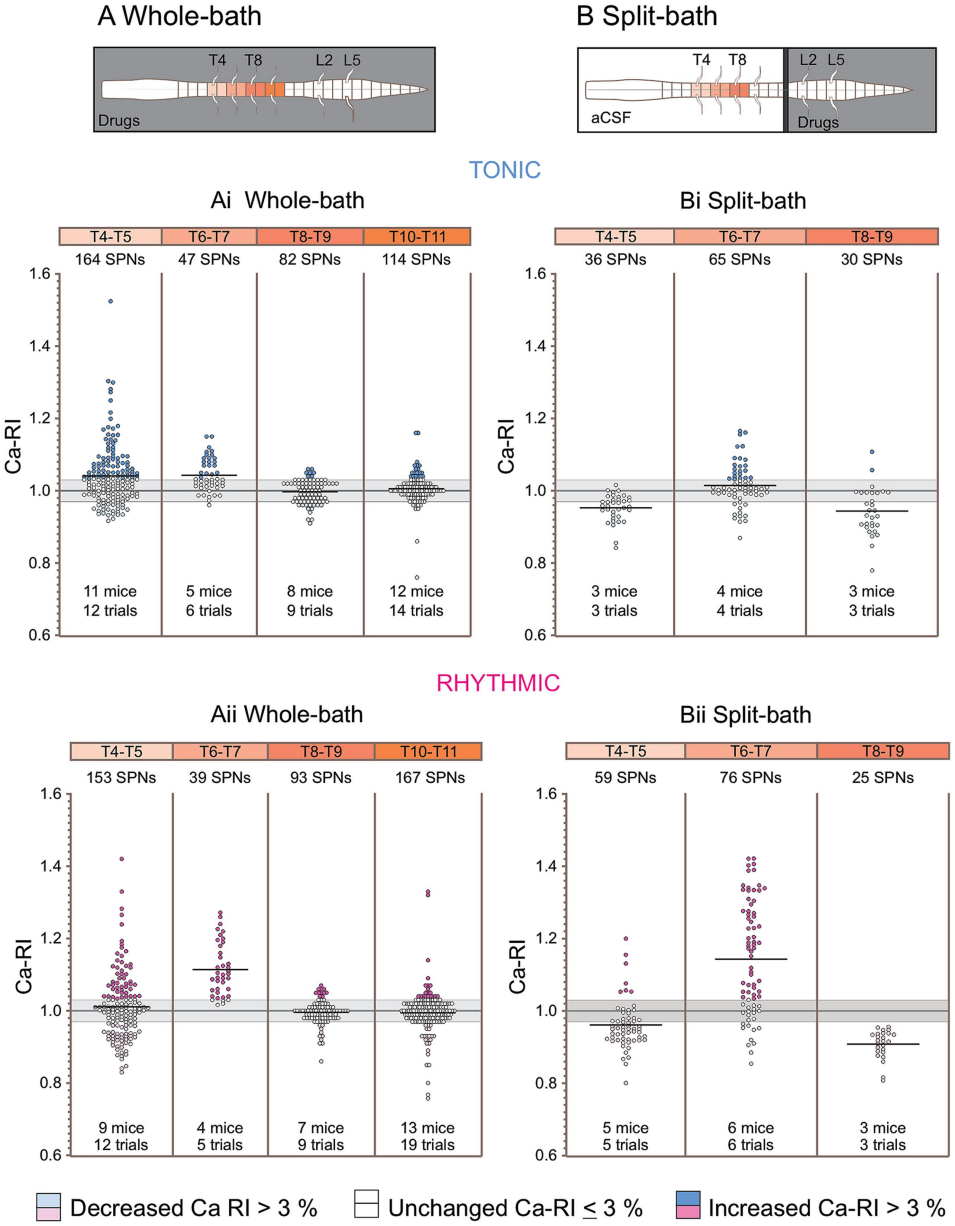
Distinct thoracic SPN Ca-RI responses are observed at different rostrocaudal levels during locomotor activity evoked by either whole cord or lumbar-only drug application. Schematic of the whole-bath design **(A)** and split-bath design for lumbar only drug application **(B)**. Ca-RI changes are summarized for all SPNs recorded at each segmental level examined (color-coded by segment to match schematics). SPN responses to whole bath application are shown on the left and split-bath application of drugs to lumbar-only regions on the right, with responses during tonic VR shown on the top and rhythmic VR activity on the bottom. The gray line is the normalized baseline (1.0), and the gray shadowed region indicates Ca-RI changes < or > 3%, with SPNs in this range depicted by clear circles. During tonic VR activity **(Ai,Bi)**, SPNs showing increases or decreases in Ca-RI > 3% are depicted by dark or light blue circles, respectively. **(Aii,Bii)**: During rhythmic VR activity, SPNs showing increases or decreases in Ca-RI > 3% are depicted by dark or light pink circles, respectively. In the whole-bath configuration during either tonic or rhythmic VR activity, greater numbers of SPNs had increased Ca-RI at rostral thoracic levels (T4 –T7) versus caudal levels (T811) **(Ai,Aii)**. Note greater increases in mean SPN intensity at T6-T7 during rhythmic VR activity (compare **Aii** to **Ai**). Similar trends were observed in SPNs with lumbar-only application of neurochemicals yet with less activation of SPNs during tonic VR activity when compared to whole cord application of drugs (compare **Bi** to **Ai**). During rhythmic VR activity in the split-bath configuration **(Bii)** there was a larger increase in SPN Ca-RI at mid-thoracic (T6-7) levels and a greater reduction in SPN Ca-RI at caudal thoracic (T8-9) levels (compare **Bii** to **Aii**). Also, with lumbar-only drug application, excitability of SPNs at more rostral (T4-T5) levels did not show similar increases in Ca-RI as that seen for whole bath application during either rhythmic or tonic activity (compare **Bii** and **Bi** to **Aii** and **Ai**).

In summary, in the split-bath configuration (Figure 6, right), at T4-5, we recorded Ca-RI in 36 SPNs during tonic and in 59 SPNs during rhythmic VR activity. During tonic activity at T4-5, Ca-RI in SPNs was either unchanged (47%, 17/36) or decreased (53%, 19/36). During rhythmic activity at T4-5, Ca-RI was increased in a minority of SPNs (12%, 7/59) whereas the majority were either unchanged [24%, 14/59 or had decreased Ca-RI (64%, 38/59)]. We recorded 65 SPNs at T6-7 during tonic VR activity, and approximately one-third (34%, 22/65) showed increased Ca-RI, most remained unchanged (48%, 31/65), and a small portion of SPNs had decreased Ca-RI (18%, 12/65). We recorded Ca-RI from 76 T6-7 SPNs during rhythmic VR activity and Ca-RI was increased in the majority of SPNs 53/76 (70%), and was either unchanged (13/76, 17%) or decreased (10/76, 13%) in the remainder of SPNs. At T8-9, Ca-RI was increased in only 2/30 (7%) of SPNs during tonic activity, remaining unchanged (30%, 9/30) or decreased (63%, 19/30) in the majority of SPNs. During rhythmic VR activity, 100% of recorded SPNs at T8-9 (25/25) showed decreased Ca-RI.

### Comparison of rostrocaudal spatial distribution of ca-RI responses in thoracic SPNs during whole- and split-bath application of neurochemicals

**Figure 7:**
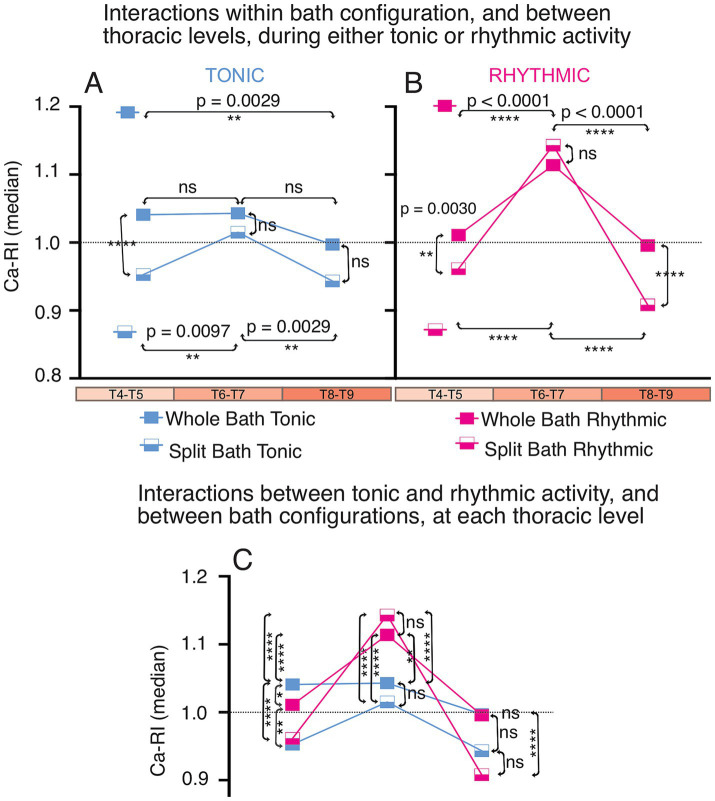
3-way ANOVA graphic showing main interactions for mean Ca-RI as the measured variable and nominative variables thoracic level (T4–T5, T6–T7, T8–T9), lumbar VR activity (tonic, rhythmic) and bath configuration (whole, split). Main interactions within bath configuration, and between thoracic region, during either tonic **(A)** or rhythmic **(B)** lumbar VR activity. Main interactions between tonic and rhythmic activity, and between bath configurations at each thoracic level is shown in **(C)**. Median values are plotted for the whole-bath (solid rectangle) and split-bath (half-filled rectangle) configurations.

Results from the whole and split-bath series of experiments are summarized and can be compared in Figure 6. Significant interactions from a 3-way ANOVA examining median Ca-RI at each thoracic level, in either the split or whole-bath configuration during tonic and rhythmic activity are shown in Figure 7 (all interactions are listed in Supplementary Table 1). Overall trends in the SPN responses at each thoracic spinal level were similar between the whole-bath and split-bath series of experiments. As shown, the greatest proportion of thoracic SPNs exhibiting an increase in Ca-RI during either tonic or rhythmic lumbar VR activity were located at the T6-7 level. This was particularly evident during rhythmic activity for both bath configurations (Figure 6 lowest panel and Figure 7B). During rhythmic activity, although a greater proportion of SPNs at T6-7 had increased Ca-RI in the whole cord versus split-bath configuration (87%, 34/39 in Figure 5Aii—whole vs. 70%, 53/76 in Figure 5Bii—split), the mean of all increased Ca-RI was greater in the split-bath configuration (1.22 ± 0.11 [1.03–1.42] in lumbar-only *versus* 1.13 ± 0.06 [1.04–1.27] in whole-cord application). However, our 3-way ANOVA analysis indicated that median Ca-RI for all SPNs recorded at T6-T7 were not significantly different when comparing split to whole bath drug application during either tonic or rhythmic (Figures 7A,B) activity. For both split and whole bath drug application, median Ca-RI during rhythmic activity was significantly higher at T6-T7 compared to either T4-T5 or T8-T9 (Figure 7B).

Comparing multiplicity-adjusted statistical interactions between thoracic segmental levels (Figure 7), indicate that *except at T6-T7*, median Ca-RI from all SPNs is decreased compared to baseline during either tonic or rhythmic activity in the split-bath configuration (Figures 6Bi,Bii, 7A,B). In contrast, whole bath application indicates median values of Ca-RI at or above baseline during both tonic and rhythmic activity at all thoracic segments examined (Figures 6Ai,Aii, 7A,B). These observations could be explained by direct effect(s) of the neurotransmitter receptor agonists used to elicit locomotor-like activity on thoracic SPNs, since each of DA, 5HT and NMDA have been reported to depolarize SPNs in slice *in vitro* (Spanswick and Logan, 1990; Pickering et al., 1994; Gladwell and Coote, 1999b; Zimmerman et al., 2012). Additionally, ANOVA analysis indicated increases in median Ca-RI were significant only at T4-T5 during tonic activity when comparing whole to split-bath drug application. No significant interactions were seen between thoracic levels for the whole bath application during tonic activity, although a peak was observed at T6-T7 for the split-bath configuration, when compared to SPNs at either T4-5 or T8-9, which either remained unchanged or decreased compared to baseline (Figures 6Bi, 7A).

Chi-square analysis indicated that the likelihood of these response patterns being due to chance for each of the whole-bath and split-bath configurations, during either tonic or rhythmic activity was extremely low, *p* < 0.0001 (Contingency tables can be seen in Supplementary data, Supplementary Figure 1).

## Discussion

The goal of this study was to examine whether activity within lumbar spinal locomotor networks can increase activity in thoracic SPNs. We previously demonstrated a direct ascending excitatory connection from a class of lumbar locomotor-related V3 INs to thoracic SPNs. We hypothesized that, similar to the integration seen at the brainstem level (Koba et al., 2022), spinal neurons are also capable of integrating locomotor network activity with sympathetic networks (SPNs), likely mediated from ascending lumbar propriospinal neurons. To achieve this, we recorded lumbar ventral root activity and calcium intensity from thoracic SPNs in an *in vitro* isolated spinal cord preparation. This preparation also allowed us to characterize SPN responses during locomotor activity in the absence of descending commands and afferent input. SPN responses were characterized by rostrocaudal segmental level, in either the presence of locomotor-inducing neurochemicals applied to the entire spinal tissue, or when applied only to the lumbar spinal region. We observed a distinctive pattern in SPN responses at different thoracic segmental levels, with mixed responses in rostral thoracic segments, increased excitability in SPNs at T6-7 and decreased excitability in SPNs at caudal segmental levels. Distinct responses in SPNs at different segments likely relates to their projection patterns and function of their ultimate target tissue or organ, as has been seen in response to vestibular nerve stimulation in which greater SPN responses were seen in T10/T12 when compared to SPNs in T2/T4 (Kasumacic et al., 2012).

### Neurotransmitter agonists elicit distinct responses in SPNs, at different thoracic segments

Known rostrocaudal differences in receptors and axon terminal projections may contribute to the differences we observed in SPN excitability at different thoracic levels, particularly during whole-bath drug application. As reviewed in Llewellyn-Smith, 2009, quantitative ultrastructural studies demonstrated that approximately 95% of the axons that provide synaptic input to SPNs contain either glutamate or GABA, although other neurotransmitters and peptides are thought to co-localize with these fast signaling amino acids (Llewellyn-Smith et al., 1992; Llewellyn-Smith et al., 1995; Llewellyn-Smith et al., 1998; Llewellyn-Smith, 2009). NMDA, 5HT and DA have all been reported to depolarize SPNs either directly or indirectly (Spanswick and Logan, 1990; Pickering et al., 1994; Gladwell and Coote, 1999a, 1999b; Zimmerman et al., 2012). In *in vitro* studies, glutamate activates SPNs through NMDA and non-NMDA receptors (Spanswick and Logan, 1990). Whereas activation of SPNs with NMDA is simply excitatory, the effects of DA and 5-HT are more complex. In response to bath-applied DA, SPNs in mid to upper thoracic spinal cord respond with either a slow hyperpolarization (at ~95 s, 46%), a slow depolarization (at ~65 s, 28%) or a biphasic response (slow hyperpolarization followed by a depolarization, or vice versa, ~33%) (Gladwell and Coote, 1999a, 1999b). The hyperpolarization and depolarization in response to DA was blocked by D_1_ and D_2_ antagonists, respectively (Gladwell and Coote, 1999a, 1999b). In caudal segments (T8-L2), DA generally increased excitability in recorded SPNs (5/7) but hyperpolarization and decreased excitability was also noted (2/7 SPNs) (Zimmerman et al., 2012). DA-containing terminals originating from the ipsilateral A11 nucleus are seen at all spinal levels, with particularly dense projections at rostral and caudal thoracic levels, terminating in the IML, around the central canal and in lamina VII (Skagerberg and Lindvall, 1985; Yoshida and Tanaka, 1988). Similarly, the effect of exogenous 5-HT application on SPNs is mixed, generally increased excitability characterized by slow and long lasting depolarizations or increased firing rates in >90% of SPNs in slice, and it can induce rhythmic oscillations, or increase the amplitude of spontaneous oscillations (without reported effects on firing rates) in SPNs from whole cord preparations (Pickering et al., 1994; Pierce et al., 2010; Zimmerman et al., 2012). Variations in the density of descending 5-HT projections at different segmental levels within thoracic IML have also been observed in multiple species [as reviewed by Jensen et al., 1995], with sympathetic nuclei in rostral and caudal thoracic regions receiving more 5-HT fibers than mid-thoracic segments (T6 – T10) (Newton and Hamill, 1988; Newton and Hamill, 1989). Interestingly, we observed a peak in median Ca-RI from SPNs in mid-thoracic compared to either rostral or caudal thoracic levels during tonic and rhythmic activity in the split-bath, and during rhythmic activity in the whole-bath application. Overall, the trends in whole- versus lumbar-bath application on SPN CaRI were similar, with an additional increase in rostral (T4-T5) thoracic SPNs Ca-RI during tonic activity. This likely reflects initial direct effects of the neurochemicals on SPNs in this region, followed later by additional indirect effects once lumbar locomotor circuitry is activated.

Thus, SPNs and related autonomic neurons within the IML are directly sensitive to exogenous NMDA, 5HT and DA application, and the responses seen during whole bath application may vary, depending upon rostro-caudal segmental level, receptor subtypes expressed on distinct populations of SPNs, in addition to any ongoing functional activity within locomotor or other neural circuits. For example, we noted that during tonic activity with whole-bath application of drugs, a greater proportion of SPNs had increased Ca-RI, and overall mean changes in Ca-RI were increased in when compared to split-bath (lumbar restricted) drug application (compare median Ca-Ri for each level examined in Figure 7). This suggests that a direct neurotransmitter effect(s) on SPNs contributed to the increased Ca-RI seen during tonic VR activity with whole-cord drug application. However, the diverse responses to the same neurotransmitter even within the same segment, likely reflects functional diversity within SPN populations and their diversity of targets, which may also contribute to the range of responses we observed in this study, including within the same thoracic segment or SPN cluster.

In this study, the T5-T6 levels showed the greatest increase in Ca-RI during tonic and rhythmic activity, either in the whole or split bath configuration. As reviewed by Deuchars and Lall (2015) SPNs in T5 and T6 provide input to the atrial and ventricular myocardium, brown adipose tissue, the adrenal and mammary glands, the kidney, spleen and ovary. Input to the myocardium and brown fat ends by ~T6/7, whereas input to kidneys, spleen and ovaries extends caudally until ~T12/13, and input to the sexual organs, bladder and bowel emerges at caudal thoracic levels (starting ~T10/11) (Strack et al., 1988; Deuchars and Lall, 2015). From a functional perspective, one might expect excitatory ascending projections from locomotor-related circuitry to preferentially increase activity in SPNs providing input to tissues and organs whose activity increases during locomotion and exercise (e.g., heart, adrenal glands or blood vessel smooth muscle to increase BP). At the same time, SPNs providing input to organs and tissues whose activity decreases during movement and exercise (i.e., kidneys, spleen, ovaries, bladder bowel, etc.,) might show reductions in Ca-RI compared to baseline (Cowley, 2018).

There is clear evidence that neural pathways that control descending sympathetic outflow are organized in the medulla and spinal cord on the basis of function (Llewellyn-Smith et al., 1998). Thus, it would seem reasonable that the pattern of ascending input to SPNs during lumbar locomotor activity would be generally reinforcing or follow a pattern of exciting/increasing activity in SPNs projecting to tissues and organs based on function as well. Our findings suggest that during locomotor activity, ascending intraspinal input to SPNs may also be functionally organized, possibly with increased excitatory input to SPNs that provide input to tissues needed when the ‘fight or flight’ arm of the autonomic nervous system is activated (e.g., vasoconstrictors to increase blood pressure). Such intraspinal organization of ascending inputs may include a component that simultaneously decreases excitatory input or inhibits tissues and organs that are considered to form part of the ‘rest and digest’ arm (e.g., decreasing intestinal motility, urine production). This research provides a proof of concept of this idea, and future studies are needed to identify specific functions associated with these patterns of ascending intraspinal input from lumbar locomotor circuits to thoracic SPNs.

### Ascending intraspinal mechanisms for integration within and between locomotor and autonomic systems

Previous studies have shown that lumbar V3 INs exhibit both ascending and descending commissural projections during embryonic development (Blacklaws et al., 2015; Deska-Gauthier et al., 2020). Although most V3 INs eventually become local commissural neurons, some evidence suggests that a subset located in the deep dorsal horn extend long ascending projections to cervical CPGs (Zhang et al., 2022). Moreover, lumbar spinal circuits have been shown to modulate the activity of reticulospinal neurons during locomotion from birth, indicating the presence of ascending projections from lumbar locomotor CPGs (Oueghlani et al., 2018). Supporting this, studies in neonatal rodent preparations demonstrated that ascending locomotor pathways can evoke locomotor-like activity in more rostral spinal segments (Juvin et al., 2005; Cherniak et al., 2014; Anglister et al., 2017). In experiments investigating the effects of cholinergic receptor agonists on both lumbar MNs and thoracic SPNs, showed that both are activated by muscarinic cholinergic agonists, suggesting there may be common neurotransmitter effects/pathways activating both systems within the spinal cord (Sourioux et al., 2018). In terms of descending intraspinal contributions, cervical CPGs are less effective at generating rhythmic activity in lumbar segments (Cowley et al., 2008; Juvin et al., 2012). Notably, Morin and colleagues proposed that both excitatory and inhibitory ascending propriospinal pathways are required for proper interlimb coordination during locomotion, based on their findings that excitatory input alone was insufficient to produce alternating rhythmic patterns (Juvin et al., 2005). Similarly, we observed increased Ca-RI at T6-T7 and decreased Ca-RI at T4-5 and T8-9 SPNs, particularly in the split-bath configuration, which suggests that both ascending excitatory and inhibitory components may contribute to regulation of sympathetic output during lumbar locomotor activity, and in a segment-specific manner. Thus, it is also possible that rhythm generation depends upon propriospinal connections between locomotor-generating and sympathetic output neurons.

### Limitations and future considerations

In our split-bath experiments, the most caudal segment we recorded SPN activity at was T9 and the most rostral segment T4. Based on the size of our microscope objective, and the location of the barrier, we were unable to record SPNs caudal to T9, and we were therefore unable to make comparisons between the whole- and split-bath drug application on SPNs located caudal to T9. It was unexpected that SPNs in T4 and T5 showed lower Ca-RI than SPNs in T6 and T7, given the large proportion of cardiac projecting SPNs in rostral thoracic segments and that one would anticipate SPNs in these segments to show increased Ca-RI. However, it is unknown if the lower Ca-RI in SPNs at T4 and T5 was related to the overall distribution pattern of all SPN projections at T6 and T7, which for example, has a peak in input to the adrenal glands (Strack et al., 1988). In future, it would be of interest to examine SPNs within rostral (T1-T3) segments as well. We previously demonstrated that at least one class of locomotor-related lumbar spinal neurons (i.e., V3 INs) provide direct synaptic excitatory input to SPNs, indicating the presence of classical excitatory amino acid synaptic communication elements between locomotor and sympathetic neural circuits in the spinal cord (Chacon et al., 2023). Future experiments recording from SPNs directly, while using specific neuron or receptor blockers will allow assessment of different receptor or cell-type contributions to SPN responses described here. Split-bath preparations and thoracic application of gap junction blockers could be used to determine any contributions of gap junctions to SPN responses reported here.

## Conclusion

These findings indicate the presence of functional intraspinal communication elements between spinal locomotor and sympathetic systems. Such coordinating communication likely contributes to increased activation of supportive homeostatic and metabolic body tissues and organs (e.g., increased BP or lipolysis). For example, a potential role(s) for this communication pathway may be to provide an ascending spinal component that contributes to the ‘exercise pressor reflex’ in which cutaneous and muscle afferents of the lower limb increase respiratory and cardiovascular activity (Coote et al., 1971; McCloskey and Mitchell, 1972), or perhaps more general coordination between rhythmic motor and sympathetic activity, as seen in the *in vivo* spinal L-Dopa adult cat or *ex vivo* adult mouse preparation (Chizh et al., 1998; Schomburg et al., 2003). We also observed decreases in activation of SPNs in caudal thoracic segments, which may relate to decreased sympathetic drive to tissues and organs whose activity is normally decreased during movement and exercise (e.g., digestion, urine production, pelvic organ function). Further research would determine whether the decreased excitation of SPNs we observed in more caudal thoracic SPNs during lumbar locomotor activity contributes to these decreases. Ascending intraspinal communication between lumbar locomotor and thoracic sympathetic circuitry may also serve to reinforce integration between ongoing movement and activation of related homeostatic and metabolic sympathetic target tissues and organs normally activated by descending central autonomic commands during movement and exercise. Together, this ascending intraspinal communication could serve to assist in the coordinated excitation and inhibition of body functions needed to sustain ongoing movement at a range of intensities and durations.

Our split-bath results suggest SPN calcium responses can be evoked through activation of lumbar CPGs, indicating that spinal locomotor networks have the ability to modulate SPN activity via ascending propriospinal connections. While descending motor and autonomic control is dominant in intact systems, our findings suggest that ascending propriospinal input contributes to modulating SPN activity during locomotion, particularly in the absence of supraspinal or sensory input. Hence, we propose that locomotor-sympathetic intraspinal pathways contribute to an integration system that regulates sympathetic outflow during locomotion and functions as a compensatory feedback mechanism to help provide or ensure adequate homeostatic support during locomotion. A recent clinical review identified several studies reporting that lumbar electrical stimulation to induce standing and stepping also improved acute sympathetic responses in a subset of metabolic and homeostatic systems (Flett et al., 2022). The improvements were only observed in subsets of people with SCI who, because of their level of injury (particularly above T1), have impaired ability to activate these systems during movement and exercise, supporting the possibility that somato-sympathetic neural spinal mechanisms may contribute to these reported outcomes (Flett et al., 2022). Understanding the neural mechanisms and contributions of pathways involved, such as we demonstrate here, may inform future therapeutic interventions specifically targeted to restore or control below-injury autonomic and motor functions after SCI.

## Data Availability

The raw data supporting the conclusions of this article will be made available by the authors, without undue reservation.

## Glossary

5-HT: Serotonin
aCSF: Artificial cerebrospinal fluid
Ca-FI: Calcium fluorescence intensity
Ca-RI: Calcium relative intensity
CDGA: Calcium dextran green amine
CPG: Central pattern generator
DA: Dopamine
EAA: Excitatory amino acid
IML: Intermediolateral cell column
IN: interneuron
INs: interneurons
MLR: Mesencephalic locomotor region
MNs: Motoneurons
NMDA: n-methyl-D-aspartate
P: postnatal
ROI: Regions of interest
RF: Reticular formation
RVLM: Rostro-ventral lateral medulla
SPNs: Sympathetic preganglionic neurons
T: thoracic
VR: Ventral root
