# Supplementary material for: Ascending propriospinal modulation of thoracic sympathetic preganglionic neurons during lumbar locomotor activity
Source: Front Neural Circuits. 2026 Mar 20;20:1738731. doi: 10.3389/fncir.2026.1738731 (PMC13047744; doi:10.3389/fncir.2026.1738731)
Supplement: Supplementary file 1 [file Data_Sheet_1.PDF]

Table Analyzed	Grouped: Entering averaged data
Three-way ANOVA	Ordinary
Alpha	0.05

Source of Variation	% of total variation	P value	P value summary	Significant?
Segmental Level Row factor	18.7	<0.0001	****	Yes
Tonic vs Rhythmic (AB vs CD)	1.084	0.0002	***	Yes
Whole Bath vs Split Bath (AC vs BD)	4.221	<0.0001	****	Yes
Segmental Level Row factor x Tonic vs Rhythmic (AB vs CD)	5.771	<0.0001	****	Yes
Segmental Level Row factor x Whole Bath vs Split Bath (AC vs BD)	2.205	<0.0001	****	Yes
Tonic vs Rhythmic (AB vs CD) x Whole Bath vs Split Bath (AC vs BD)	0.2102	0.1038	ns	No
Segmental Level Row factor x Tonic vs Rhythmic (AB vs CD) x Whole Bath vs Split Bath (AC vs BD)	0.6565	0.0163	*	Yes

ANOVA table	SS (Type III)	DF	MS	F (DFn, DFd)	P value
Segmental Level Row factor	1.493	2	0.7464	F (2, 857) = 117.9	P<0.0001
Tonic vs Rhythmic (AB vs CD)	0.08653	1	0.08653	F (1, 857) = 13.67	P=0.0002
Whole Bath vs Split Bath (AC vs BD)	0.3368	1	0.3368	F (1, 857) = 53.23	P<0.0001
Segmental Level Row factor x Tonic vs Rhythmic (AB vs CD)	0.4606	2	0.2303	F (2, 857) = 36.39	P<0.0001
Segmental Level Row factor x Whole Bath vs Split Bath (AC vs BD)	0.176	2	0.08799	F (2, 857) = 13.90	P<0.0001
Tonic vs Rhythmic (AB vs CD) x Whole Bath vs Split Bath (AC vs BD)	0.01678	1	0.01678	F (1, 857) = 2.651	P=0.1038
Segmental Level Row factor x Tonic vs Rhythmic (AB vs CD) x Whole Bath vs Split Bath (AC vs BD)	0.05239	2	0.0262	F (2, 857) = 4.139	P=0.0163
Residual	5.424	857	0.00633		

3 way ANOVA Multiple Comparisons

	Whole Bath Tonic			Split Bath Tonic		
Segment	Mean	SD	N	Mean	SD	N
T4-T5	1.041	0.08364	164	0.9528	0.03801	36
T6-T7	1.043	0.04589	47	1.015	0.05856	65
T8-T9	0.9971	0.03256	82	0.9434	0.066	30

	Whole Bath Rhythmic			Split Bath Rhythmic		
	Mean	SD	N	Mean	SD	N
T4-T5	1.011	0.09232	153	0.9614	0.06831	59
T6-T7	1.114	0.07331	39	1.143	0.1544	76
T8-T9	0.9955	0.03444	93	0.9082	0.03919	25

Number of families	1
Number of comparisons per family	66
Alpha	0.05

Tukey's multiple comparisons test	Mean diff.	95.00% CI of diff.	Below threshold?	Summary	Adjusted P Value
T4-T5:Whole Bath Tonic vs. T4-T5:Split Bath Tonic	0.0882	0.04022 to 0.1362	Yes	****	<0.0001
T4-T5:Whole Bath Tonic vs. T4-T5:Whole Bath Rhythmic	0.03	0.0006969 to 0.05930	Yes	*	0.0393
T4-T5:Whole Bath Tonic vs. T4-T5:Split Bath Rhythmic	0.0796	0.04002 to 0.1192	Yes	****	<0.0001
T4-T5:Whole Bath Tonic vs. T6-T7:Whole Bath Tonic	-0.002	-0.04513 to 0.04113	No	ns	>0.9999
T4-T5:Whole Bath Tonic vs. T6-T7:Split Bath Tonic	0.026	-0.01221 to 0.06421	No	ns	0.5282
T4-T5:Whole Bath Tonic vs. T6-T7:Whole Bath Rhythmic	-0.073	-0.1194 to -0.02655	Yes	****	<0.0001
T4-T5:Whole Bath Tonic vs. T6-T7:Split Bath Rhythmic	-0.102	-0.1382 to -0.06582	Yes	****	<0.0001
T4-T5:Whole Bath Tonic vs. T8-T9:Whole Bath Tonic	0.0439	0.008639 to 0.07916	Yes	**	0.0029
T4-T5:Whole Bath Tonic vs. T8-T9:Split Bath Tonic	0.0976	0.04583 to 0.1494	Yes	****	<0.0001
T4-T5:Whole Bath Tonic vs. T8-T9:Whole Bath Rhythmic	0.0455	0.01166 to 0.07934	Yes	***	0.0007

T4-T5:Whole Bath Tonic vs. T8-T9:Split Bath Rhythmic	0.1328	0.07683 to 0.1888	Yes	****	<0.0001
T4-T5:Split Bath Tonic vs. T4-T5:Whole Bath Rhythmic	-0.0582	-0.1065 to -0.009907	Yes	**	0.0048
T4-T5:Split Bath Tonic vs. T4-T5:Split Bath Rhythmic	-0.0086	-0.06374 to 0.04654	No	ns	>0.9999
T4-T5:Split Bath Tonic vs. T6-T7:Whole Bath Tonic	-0.0902	-0.1479 to -0.03246	Yes	****	<0.0001
T4-T5:Split Bath Tonic vs. T6-T7:Split Bath Tonic	-0.0622	-0.1164 to -0.008037	Yes	**	0.0097
T4-T5:Split Bath Tonic vs. T6-T7:Whole Bath Rhythmic	-0.1612	-0.2215 to -0.1009	Yes	****	<0.0001
T4-T5:Split Bath Tonic vs. T6-T7:Split Bath Rhythmic	-0.1902	-0.2429 to -0.1375	Yes	****	<0.0001
T4-T5:Split Bath Tonic vs. T8-T9:Whole Bath Tonic	-0.0443	-0.09642 to 0.007824	No	ns	0.1881
T4-T5:Split Bath Tonic vs. T8-T9:Split Bath Tonic	0.0094	-0.05505 to 0.07385	No	ns	>0.9999
T4-T5:Split Bath Tonic vs. T8-T9:Whole Bath Rhythmic	-0.0427	-0.09387 to 0.008474	No	ns	0.2112
T4-T5:Split Bath Tonic vs. T8-T9:Split Bath Rhythmic	0.0446	-0.02327 to 0.1125	No	ns	0.5841
T4-T5:Whole Bath Rhythmic vs. T4-T5:Split Bath Rhythmic	0.0496	0.009647 to 0.08955	Yes	**	0.003
T4-T5:Whole Bath Rhythmic vs. T6-T7:Whole Bath Tonic	-0.032	-0.07548 to 0.01148	No	ns	0.3989
T4-T5:Whole Bath Rhythmic vs. T6-T7:Split Bath Tonic	-0.004	-0.04260 to 0.03460	No	ns	>0.9999
T4-T5:Whole Bath Rhythmic vs. T6-T7:Whole Bath Rhythmic	-0.103	-0.1498 to -0.05623	Yes	****	<0.0001
T4-T5:Whole Bath Rhythmic vs. T6-T7:Split Bath Rhythmic	-0.132	-0.1686 to -0.09541	Yes	****	<0.0001
T4-T5:Whole Bath Rhythmic vs. T8-T9:Whole Bath Tonic	0.0139	-0.02178 to 0.04958	No	ns	0.9818
T4-T5:Whole Bath Rhythmic vs. T8-T9:Split Bath Tonic	0.0676	0.01554 to 0.1197	Yes	**	0.0014
T4-T5:Whole Bath Rhythmic vs. T8-T9:Whole Bath Rhythmic	0.0155	-0.01878 to 0.04978	No	ns	0.9454
T4-T5:Whole Bath Rhythmic vs. T8-T9:Split Bath Rhythmic	0.1028	0.04656 to 0.1590	Yes	****	<0.0001
T4-T5:Split Bath Rhythmic vs. T6-T7:Whole Bath Tonic	-0.0816	-0.1326 to -0.03063	Yes	****	<0.0001
T4-T5:Split Bath Rhythmic vs. T6-T7:Split Bath Tonic	-0.0536	-0.1005 to -0.006721	Yes	*	0.0103
T4-T5:Split Bath Rhythmic vs. T6-T7:Whole Bath Rhythmic	-0.1526	-0.2064 to -0.09880	Yes	****	<0.0001
T4-T5:Split Bath Rhythmic vs. T6-T7:Split Bath Rhythmic	-0.1816	-0.2268 to -0.1364	Yes	****	<0.0001
T4-T5:Split Bath Rhythmic vs. T8-T9:Whole Bath Tonic	-0.0357	-0.08021 to 0.008807	No	ns	0.2652
T4-T5:Split Bath Rhythmic vs. T8-T9:Split Bath Tonic	0.018	-0.04046 to 0.07646	No	ns	0.9975
T4-T5:Split Bath Rhythmic vs. T8-T9:Whole Bath Rhythmic	-0.0341	-0.07749 to 0.009292	No	ns	0.2954
T4-T5:Split Bath Rhythmic vs. T8-T9:Split Bath Rhythmic	0.0532	-0.009015 to 0.1154	No	ns	0.1808
T6-T7:Whole Bath Tonic vs. T6-T7:Split Bath Tonic	0.028	-0.02192 to 0.07792	No	ns	0.7964
T6-T7:Whole Bath Tonic vs. T6-T7:Whole Bath Rhythmic	-0.071	-0.1275 to -0.01453	Yes	**	0.0024
T6-T7:Whole Bath Tonic vs. T6-T7:Split Bath Rhythmic	-0.1	-0.1484 to -0.05162	Yes	****	<0.0001
T6-T7:Whole Bath Tonic vs. T8-T9:Whole Bath Tonic	0.0459	-0.001797 to 0.09360	No	ns	0.0721
T6-T7:Whole Bath Tonic vs. T8-T9:Split Bath Tonic	0.0996	0.03868 to 0.1605	Yes	****	<0.0001
T6-T7:Whole Bath Tonic vs. T8-T9:Whole Bath Rhythmic	0.0475	0.0008422 to 0.09416	Yes	*	0.0416
T6-T7:Whole Bath Tonic vs. T8-T9:Split Bath Rhythmic	0.1348	0.07026 to 0.1993	Yes	****	<0.0001

T6-T7:Split Bath Tonic vs. T6-T7:Whole Bath Rhythmic	-0.099	-0.1518 to -0.04619	Yes	****	<0.0001
T6-T7:Split Bath Tonic vs. T6-T7:Split Bath Rhythmic	-0.128	-0.1720 to -0.08395	Yes	****	<0.0001
T6-T7:Split Bath Tonic vs. T8-T9:Whole Bath Tonic	0.0179	-0.02540 to 0.06120	No	ns	0.9714
T6-T7:Split Bath Tonic vs. T8-T9:Split Bath Tonic	0.0716	0.01406 to 0.1291	Yes	**	0.0029
T6-T7:Split Bath Tonic vs. T8-T9:Whole Bath Rhythmic	0.0195	-0.02265 to 0.06165	No	ns	0.9361
T6-T7:Split Bath Tonic vs. T8-T9:Split Bath Rhythmic	0.1068	0.04545 to 0.1682	Yes	****	<0.0001
T6-T7:Whole Bath Rhythmic vs. T6-T7:Split Bath Rhythmic	-0.029	-0.08035 to 0.02235	No	ns	0.7891
T6-T7:Whole Bath Rhythmic vs. T8-T9:Whole Bath Tonic	0.1169	0.06619 to 0.1676	Yes	****	<0.0001
T6-T7:Whole Bath Rhythmic vs. T8-T9:Split Bath Tonic	0.1706	0.1073 to 0.2339	Yes	****	<0.0001
T6-T7:Whole Bath Rhythmic vs. T8-T9:Whole Bath Rhythmic	0.1185	0.06876 to 0.1682	Yes	****	<0.0001
T6-T7:Whole Bath Rhythmic vs. T8-T9:Split Bath Rhythmic	0.2058	0.1390 to 0.2726	Yes	****	<0.0001
T6-T7:Split Bath Rhythmic vs. T8-T9:Whole Bath Tonic	0.1459	0.1044 to 0.1874	Yes	****	<0.0001
T6-T7:Split Bath Rhythmic vs. T8-T9:Split Bath Tonic	0.1996	0.1434 to 0.2558	Yes	****	<0.0001
T6-T7:Split Bath Rhythmic vs. T8-T9:Whole Bath Rhythmic	0.1475	0.1072 to 0.1878	Yes	****	<0.0001
T6-T7:Split Bath Rhythmic vs. T8-T9:Split Bath Rhythmic	0.2348	0.1747 to 0.2949	Yes	****	<0.0001
T8-T9:Whole Bath Tonic vs. T8-T9:Split Bath Tonic	0.0537	-0.001928 to 0.1093	No	ns	0.0701
T8-T9:Whole Bath Tonic vs. T8-T9:Whole Bath Rhythmic	0.0016	-0.03789 to 0.04109	No	ns	>0.9999
T8-T9:Whole Bath Tonic vs. T8-T9:Split Bath Rhythmic	0.0889	0.02934 to 0.1485	Yes	****	<0.0001
T8-T9:Split Bath Tonic vs. T8-T9:Whole Bath Rhythmic	-0.0521	-0.1068 to 0.002640	No	ns	0.0796
T8-T9:Split Bath Tonic vs. T8-T9:Split Bath Rhythmic	0.0352	-0.03540 to 0.1058	No	ns	0.8962
T8-T9:Whole Bath Rhythmic vs. T8-T9:Split Bath Rhythmic	0.0873	0.02857 to 0.1460	Yes	****	<0.0001

Test details	Mean 1	Mean 2	Mean diff.	SE of diff.	N1	N2	q	DF
T4-T5:Whole Bath Tonic vs. T4-T5:Split Bath Tonic	1.041	0.9528	0.0882	0.01464	164	36	8.519	857
T4-T5:Whole Bath Tonic vs. T4-T5:Whole Bath Rhythmic	1.041	1.011	0.03	0.00894	164	153	4.745	857
T4-T5:Whole Bath Tonic vs. T4-T5:Split Bath Rhythmic	1.041	0.9614	0.0796	0.01208	164	59	9.321	857
T4-T5:Whole Bath Tonic vs. T6-T7:Whole Bath Tonic	1.041	1.043	-0.002	0.01316	164	47	0.215	857
T4-T5:Whole Bath Tonic vs. T6-T7:Split Bath Tonic	1.041	1.015	0.026	0.01166	164	65	3.153	857
T4-T5:Whole Bath Tonic vs. T6-T7:Whole Bath Rhythmic	1.041	1.114	-0.073	0.01417	164	39	7.284	857
T4-T5:Whole Bath Tonic vs. T6-T7:Split Bath Rhythmic	1.041	1.143	-0.102	0.01104	164	76	13.07	857
T4-T5:Whole Bath Tonic vs. T8-T9:Whole Bath Tonic	1.041	0.9971	0.0439	0.01076	164	82	5.77	857
T4-T5:Whole Bath Tonic vs. T8-T9:Split Bath Tonic	1.041	0.9434	0.0976	0.0158	164	30	8.738	857
T4-T5:Whole Bath Tonic vs. T8-T9:Whole Bath Rhythmic	1.041	0.9955	0.0455	0.01033	164	93	6.231	857

T4-T5:Whole Bath Tonic vs. T8-T9:Split Bath Rhythmic	1.041	0.9082	0.1328	0.01708	164	25	11	857
T4-T5:Split Bath Tonic vs. T4-T5:Whole Bath Rhythmic	0.9528	1.011	-0.0582	0.01474	36	153	5.585	857
T4-T5:Split Bath Tonic vs. T4-T5:Split Bath Rhythmic	0.9528	0.9614	-0.0086	0.01682	36	59	0.723	857
T4-T5:Split Bath Tonic vs. T6-T7:Whole Bath Tonic	0.9528	1.043	-0.0902	0.01762	36	47	7.24	857
T4-T5:Split Bath Tonic vs. T6-T7:Split Bath Tonic	0.9528	1.015	-0.0622	0.01653	36	65	5.322	857
T4-T5:Split Bath Tonic vs. T6-T7:Whole Bath Rhythmic	0.9528	1.114	-0.1612	0.01839	36	39	12.4	857
T4-T5:Split Bath Tonic vs. T6-T7:Split Bath Rhythmic	0.9528	1.143	-0.1902	0.0161	36	76	16.71	857
T4-T5:Split Bath Tonic vs. T8-T9:Whole Bath Tonic	0.9528	0.9971	-0.0443	0.01591	36	82	3.939	857
T4-T5:Split Bath Tonic vs. T8-T9:Split Bath Tonic	0.9528	0.9434	0.0094	0.01967	36	30	0.676	857
T4-T5:Split Bath Tonic vs. T8-T9:Whole Bath Rhythmic	0.9528	0.9955	-0.0427	0.01562	36	93	3.867	857
T4-T5:Split Bath Tonic vs. T8-T9:Split Bath Rhythmic	0.9528	0.9082	0.0446	0.02071	36	25	3.045	857
T4-T5:Whole Bath Rhythmic vs. T4-T5:Split Bath Rhythmic	1.011	0.9614	0.0496	0.01219	153	59	5.754	857
T4-T5:Whole Bath Rhythmic vs. T6-T7:Whole Bath Tonic	1.011	1.043	-0.032	0.01327	153	47	3.411	857
T4-T5:Whole Bath Rhythmic vs. T6-T7:Split Bath Tonic	1.011	1.015	-0.004	0.01178	153	65	0.48	857
T4-T5:Whole Bath Rhythmic vs. T6-T7:Whole Bath Rhythmic	1.011	1.114	-0.103	0.01427	153	39	10.21	857
T4-T5:Whole Bath Rhythmic vs. T6-T7:Split Bath Rhythmic	1.011	1.143	-0.132	0.01116	153	76	16.72	857
T4-T5:Whole Bath Rhythmic vs. T8-T9:Whole Bath Tonic	1.011	0.9971	0.0139	0.01089	153	82	1.805	857
T4-T5:Whole Bath Rhythmic vs. T8-T9:Split Bath Tonic	1.011	0.9434	0.0676	0.01588	153	30	6.018	857
T4-T5:Whole Bath Rhythmic vs. T8-T9:Whole Bath Rhythmic	1.011	0.9955	0.0155	0.01046	153	93	2.096	857
T4-T5:Whole Bath Rhythmic vs. T8-T9:Split Bath Rhythmic	1.011	0.9082	0.1028	0.01716	153	25	8.471	857
T4-T5:Split Bath Rhythmic vs. T6-T7:Whole Bath Tonic	0.9614	1.043	-0.0816	0.01555	59	47	7.419	857
T4-T5:Split Bath Rhythmic vs. T6-T7:Split Bath Tonic	0.9614	1.015	-0.0536	0.0143	59	65	5.299	857
T4-T5:Split Bath Rhythmic vs. T6-T7:Whole Bath Rhythmic	0.9614	1.114	-0.1526	0.01642	59	39	13.14	857
T4-T5:Split Bath Rhythmic vs. T6-T7:Split Bath Rhythmic	0.9614	1.143	-0.1816	0.0138	59	76	18.61	857
T4-T5:Split Bath Rhythmic vs. T8-T9:Whole Bath Tonic	0.9614	0.9971	-0.0357	0.01358	59	82	3.717	857
T4-T5:Split Bath Rhythmic vs. T8-T9:Split Bath Tonic	0.9614	0.9434	0.018	0.01784	59	30	1.427	857
T4-T5:Split Bath Rhythmic vs. T8-T9:Whole Bath Rhythmic	0.9614	0.9955	-0.0341	0.01324	59	93	3.642	857
T4-T5:Split Bath Rhythmic vs. T8-T9:Split Bath Rhythmic	0.9614	0.9082	0.0532	0.01898	59	25	3.963	857
T6-T7:Whole Bath Tonic vs. T6-T7:Split Bath Tonic	1.043	1.015	0.028	0.01523	47	65	2.6	857
T6-T7:Whole Bath Tonic vs. T6-T7:Whole Bath Rhythmic	1.043	1.114	-0.071	0.01723	47	39	5.827	857
T6-T7:Whole Bath Tonic vs. T6-T7:Split Bath Rhythmic	1.043	1.143	-0.1	0.01476	47	76	9.58	857
T6-T7:Whole Bath Tonic vs. T8-T9:Whole Bath Tonic	1.043	0.9971	0.0459	0.01455	47	82	4.46	857
T6-T7:Whole Bath Tonic vs. T8-T9:Split Bath Tonic	1.043	0.9434	0.0996	0.01859	47	30	7.577	857
T6-T7:Whole Bath Tonic vs. T8-T9:Whole Bath Rhythmic	1.043	0.9955	0.0475	0.01424	47	93	4.718	857
T6-T7:Whole Bath Tonic vs. T8-T9:Split Bath Rhythmic	1.043	0.9082	0.1348	0.01969	47	25	9.681	857

T6-T7:Split Bath Tonic vs. T6-T7:Whole Bath Rhythmic	1.015	1.114	-0.099	0.01611	65	39	8.689	857
T6-T7:Split Bath Tonic vs. T6-T7:Split Bath Rhythmic	1.015	1.143	-0.128	0.01344	65	76	13.47	857
T6-T7:Split Bath Tonic vs. T8-T9:Whole Bath Tonic	1.015	0.9971	0.0179	0.01321	65	82	1.916	857
T6-T7:Split Bath Tonic vs. T8-T9:Split Bath Tonic	1.015	0.9434	0.0716	0.01756	65	30	5.767	857
T6-T7:Split Bath Tonic vs. T8-T9:Whole Bath Rhythmic	1.015	0.9955	0.0195	0.01286	65	93	2.144	857
T6-T7:Split Bath Tonic vs. T8-T9:Split Bath Rhythmic	1.015	0.9082	0.1068	0.01872	65	25	8.067	857
T6-T7:Whole Bath Rhythmic vs. T6-T7:Split Bath Rhythmic	1.114	1.143	-0.029	0.01567	39	76	2.617	857
T6-T7:Whole Bath Rhythmic vs. T8-T9:Whole Bath Tonic	1.114	0.9971	0.1169	0.01547	39	82	10.68	857
T6-T7:Whole Bath Rhythmic vs. T8-T9:Split Bath Tonic	1.114	0.9434	0.1706	0.01932	39	30	12.49	857
T6-T7:Whole Bath Rhythmic vs. T8-T9:Whole Bath Rhythmic	1.114	0.9955	0.1185	0.01518	39	93	11.04	857
T6-T7:Whole Bath Rhythmic vs. T8-T9:Split Bath Rhythmic	1.114	0.9082	0.2058	0.02038	39	25	14.28	857
T6-T7:Split Bath Rhythmic vs. T8-T9:Whole Bath Tonic	1.143	0.9971	0.1459	0.01267	76	82	16.29	857
T6-T7:Split Bath Rhythmic vs. T8-T9:Split Bath Tonic	1.143	0.9434	0.1996	0.01715	76	30	16.46	857
T6-T7:Split Bath Rhythmic vs. T8-T9:Whole Bath Rhythmic	1.143	0.9955	0.1475	0.0123	76	93	16.96	857
T6-T7:Split Bath Rhythmic vs. T8-T9:Split Bath Rhythmic	1.143	0.9082	0.2348	0.01834	76	25	18.1	857
T8-T9:Whole Bath Tonic vs. T8-T9:Split Bath Tonic	0.9971	0.9434	0.0537	0.01697	82	30	4.474	857
T8-T9:Whole Bath Tonic vs. T8-T9:Whole Bath Rhythmic	0.9971	0.9955	0.0016	0.01205	82	93	0.188	857
T8-T9:Whole Bath Tonic vs. T8-T9:Split Bath Rhythmic	0.9971	0.9082	0.0889	0.01817	82	25	6.917	857
T8-T9:Split Bath Tonic vs. T8-T9:Whole Bath Rhythmic	0.9434	0.9955	-0.0521	0.0167	30	93	4.411	857
T8-T9:Split Bath Tonic vs. T8-T9:Split Bath Rhythmic	0.9434	0.9082	0.0352	0.02154	30	25	2.311	857
T8-T9:Whole Bath Rhythmic vs. T8-T9:Split Bath Rhythmic	0.9955	0.9082	0.0873	0.01792	93	25	6.889	857
