# Supplementary figures and images for: Ascending propriospinal modulation of thoracic sympathetic preganglionic neurons during lumbar locomotor activity
Source: Front Neural Circuits. 2026 Mar 20;20:1738731. doi: 10.3389/fncir.2026.1738731 (PMC13047744; doi:10.3389/fncir.2026.1738731)

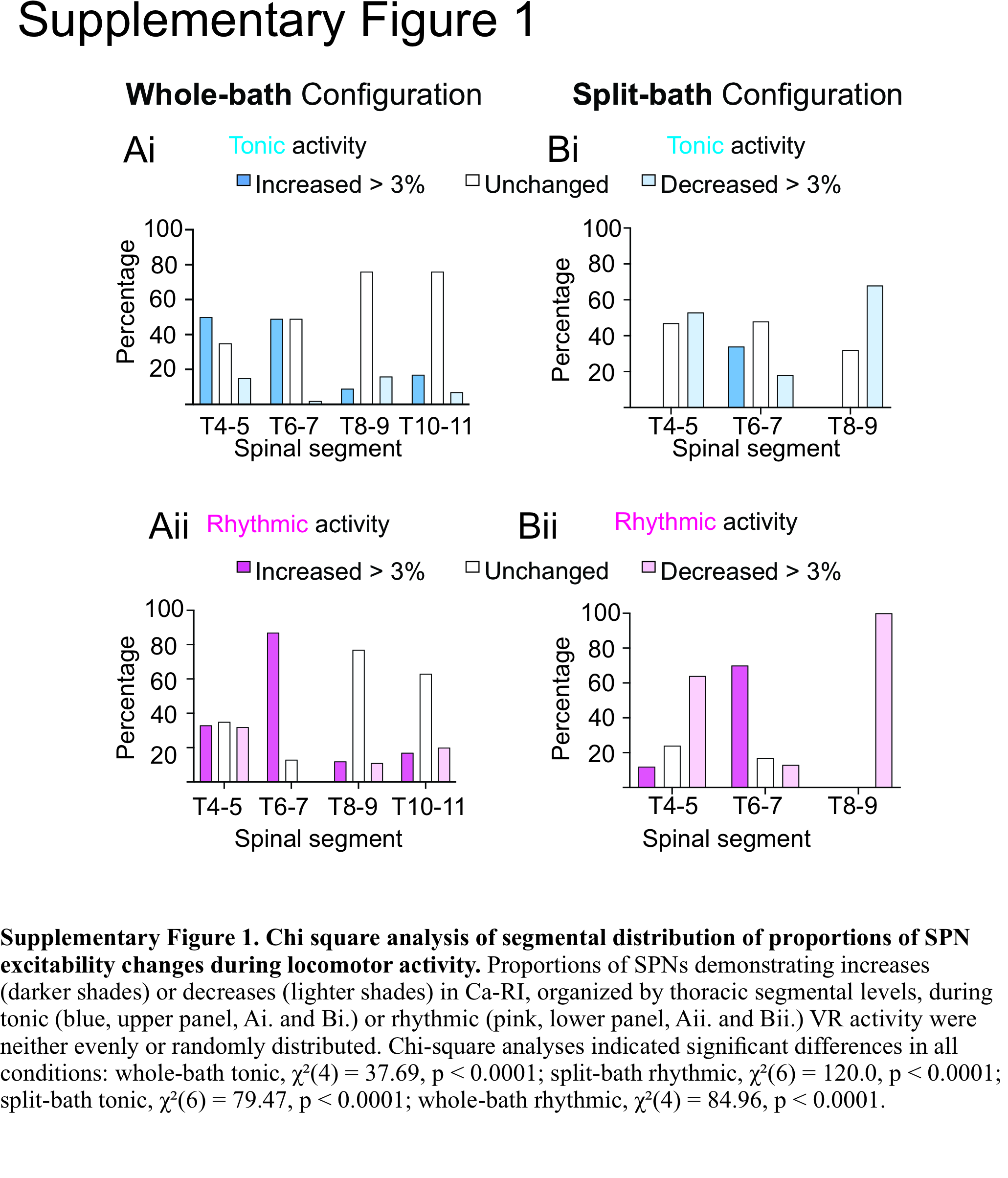

Supplement: Supplementary file 2 [file Image_1.TIF]
